# Management of gout following 2016/2017 European (EULAR) and British (BSR) guidelines: An interrupted time-series analysis in the United Kingdom

**DOI:** 10.1016/j.lanepe.2022.100416

**Published:** 2022-05-25

**Authors:** Mark D Russell, Andrew I Rutherford, Benjamin Ellis, Sam Norton, Abdel Douiri, Martin C Gulliford, Andrew P Cope, James B Galloway

**Affiliations:** a Centre for Rheumatic Diseases, King's College London, SE5 9RJ, UK; b Department of Rheumatology, King's College Hospital NHS Foundation Trust, London, SE5 9RS, UK; c Department of Rheumatology, Imperial College Healthcare NHS Foundation Trust, London; d School of Population Health and Environmental Sciences, King's College London, SE1 1UL, UK

**Keywords:** Gout, Crystal arthritis, Urate-lowering therapy, Allopurinol, Epidemiology, Treat-to-target

## Abstract

**Background:**

Following studies reporting sub-optimal gout management, European (EULAR) and British (BSR) guidelines were updated to encourage the prescription of urate-lowering therapy (ULT) with a treat-to-target approach. We investigated whether ULT initiation and urate target attainment has improved following publication of these guidelines, and assessed predictors of these outcomes.

**Methods:**

We used the Clinical Practice Research Datalink to assess attainment of the following outcomes in people (*n* = 129,972) with index gout diagnoses in the UK from 2004-2020: i) initiation of ULT; ii) serum urate ≤360 µmol/L and ≤300 µmol/L; iii) treat-to-target urate monitoring. Interrupted time-series analyses were used to compare trends in outcomes before and after updated EULAR and BSR management guidelines, published in 2016 and 2017, respectively. Predictors of ULT initiation and urate target attainment were modelled using logistic regression and Cox proportional hazards.

**Findings:**

37,529 (28.9%) of 129,972 people with newly-diagnosed gout had ULT initiated within 12 months. ULT initiation improved modestly over the study period, from 26.8% for those diagnosed in 2004 to 36.6% in 2019 and 34.7% in 2020. Of people diagnosed in 2020 with a serum urate performed within 12 months, 17.1% attained a urate ≤300 µmol/L, while 36.0% attained a urate ≤360 µmol/L. 18.9% received treat-to-target urate monitoring. No significant improvements in ULT initiation or urate target attainment were observed after updated BSR or EULAR management guidance, relative to before. Comorbidities, including chronic kidney disease (CKD), heart failure and obesity, and diuretic use associated with increased odds of ULT initiation but decreased odds of attaining urate targets within 12 months: CKD (adjusted OR 1.61 for ULT initiation, 95% CI 1.55 to 1.67; adjusted OR 0.51 for urate ≤300 µmol/L, 95% CI 0.48 to 0.55; both *p* < 0.001); heart failure (adjusted OR 1.56 for ULT initiation, 95% CI 1.48 to 1.64; adjusted OR 0.85 for urate ≤300 µmol/L, 95% CI 0.76 to 0.95; both *p* < 0.001); obesity (adjusted OR 1.32 for ULT initiation, 95% CI 1.29 to 1.36; adjusted OR 0.61 for urate ≤300 µmol/L, 95% CI 0.58 to 0.65; both *p* < 0.001); and diuretic use (adjusted OR 1.49 for ULT initiation, 95% CI 1.44 to 1.55; adjusted OR 0.61 for urate ≤300 µmol/L, 95% CI 0.57 to 0.66; both *p* < 0.001).

**Interpretation:**

Initiation of ULT and attainment of urate targets remain poor for people diagnosed with gout in the UK, despite updated management guidelines. If the evidence-practice gap in gout management is to be bridged, strategies to implement best practice care are needed.

**Funding:**

National Institute for Health Research.

**Research in context**

**Evidence before the study**

Gout is a common and highly treatable form of inflammatory arthritis, yet one of the most poorly managed. We searched PubMed up to 1^st^ February 2022 using the terms “gout and management”, to identify population-based cohort studies reporting on the prescription of urate-lowering therapy (ULT) and/or the attainment of target urate levels in people with gout. Eight studies from the United Kingdom, United States, Australia, New Zealand, Sweden and Taiwan, with data up to 2016, showed that the majority of people with gout were not prescribed ULT and did not attain target urate levels after diagnosis. Since then, the British Society for Rheumatology (BSR) and European Alliance of Associations for Rheumatology (EULAR) have updated their gout management guidelines, to encourage the prescription of ULT, and for the dose of ULT to be uptitrated until a target urate level (≤300 µmol/L or ≤360 µmol/L) is achieved.

**Added value of this study**

Our study analysed a UK cohort with 129,972 people with newly-diagnosed gout from 2004 to 2020. We showed that for people diagnosed with gout in 2020, only 35% were initiated on ULT within a year of diagnosis, while only 36% and 17% attained urate levels of ≤360 µmol/L and ≤300 µmol/L, respectively. Trends in ULT initiation and urate target attainment have not changed significantly following publication of updated gout management guidelines. People with multimorbidity at diagnosis are less likely to attain target urate levels than people without comorbidities.

**Implications of all the available evidence**

Our findings show that, despite updated guidelines, the majority of people with gout in the UK are still not being initiated on ULT, and do not attain the target urate levels necessary to prevent flares, hospitalisations, and morbidity. People with multimorbidity at diagnosis are even less likely to attain urate targets. Our findings suggest that for there to be improvements in gout care, implementation strategies to encourage the uptake of guideline-recommended treatments are urgently needed.

Alt-text: Unlabelled box

## Introduction

Gout is the most common form of inflammatory arthritis, with a prevalence of 2.5% in the UK and 3.9% of adults in the United States.^1^^,^^2^ In the context of chronic hyperuricaemia and urate crystal deposition, gout is characterised by recurrent flares of joint pain and swelling, erosive joint damage, and extra-articular sequelae such as renal impairment.

Gout is also the only curable form of inflammatory arthritis: flares are preventable with urate-lowering therapy (ULT), of which allopurinol is the first-line recommended treatment.^3^ Despite this, in 2012, only 27% of people with gout in UK primary care received prescriptions for ULT within 12 months of diagnosis.^1^ Moreover, only a minority achieve the serum urate levels necessary to prevent gout flares and morbidity.^4^^,^^5^ Studies in other countries, including the United States, Australia, New Zealand, Sweden and Taiwan have also reported sub-optimal levels of ULT initiation and target attainment.^6^, ^7^, ^8^, ^9^, ^10^, ^11^, ^12^

Recognising the need for improvement, the European Alliance of Associations for Rheumatology (EULAR) and British Society for Rheumatology (BSR) updated their gout management guidelines in 2016 and 2017, respectively.^3^^,^^13^ The BSR guideline recommends that all patients with gout should have ULT discussed and offered to them, while EULAR guidance recommends that ULT should be considered and discussed with every patient with a definite diagnosis of gout from the first presentation. The prescription of ULT is strongly encouraged in people with gout who have risk factors that include chronic kidney disease (CKD), cardiovascular comorbidities (hypertension, ischaemic heart disease (IHD) and heart failure), urolithiasis, diuretic use, or gout diagnosis at a young age.^3^^,^^13^ Once initiated, it is recommended that the dose of ULT is uptitrated to achieve a serum urate level that is below the saturation threshold, thereby preventing new crystal formation and helping to dissolve pre-existing crystals. The target urate level is ≤300 µmol/L in the BSR guideline, and ≤300 µmol/L or ≤360 µmol/L, depending on gout severity, in the EULAR guideline.^3^

Whether gout management has improved, particularly following the publication of updated guidelines, is not known. In this study, we performed analyses of people diagnosed with gout in the UK between 2004 and 2020, to assess the following objectives: i) temporal trends in the initiation of ULT, and predictors thereof; ii) trends in the implementation of a treat-to-target approach with regards to serum urate levels and monitoring; and iii) predictors of attaining target serum urate levels.

## Methods

### Data source

The Clinical Practice Research Datalink (CPRD) is a longitudinal, representative health database containing anonymised demographic, clinical and prescription data from people registered with over 2000 primary care practices in the UK.^14^ In this study, we used the CPRD GOLD dataset, containing data on over 20 million people from general practices using Vision® electronic health record software.

### Study population and case definition

We conducted a population-level, observational cohort study of people aged ≥18 years, currently or previously registered with a CPRD GOLD practice, with index gout diagnoses between 1^st^ January 2004 and 21^st^ October 2020. The start date of 2004 corresponds to the more widespread availability of laboratory-linked data with the incorporation of the Quality and Outcomes Framework into UK primary care contracts.

An index gout diagnosis was defined as a new diagnostic code for incident gout in people without previous gout diagnostic codes (see supplementary appendix for included Read codes). A minimum of 12 months of registration with a CPRD practice prior to the first gout diagnostic code was required to ensure only incident cases were detected, in addition to a minimum of 12 months of follow-up post-diagnosis.

### Outcomes and predictor variables

Primary outcome measures assessed were: i) a new prescription for ULT (allopurinol, febuxostat, benzbromarone, probenecid or sulfinpyrazone) within 12 months of the index gout diagnosis date; ii) a recorded serum urate level ≤360 µmol/L within 12 months of index diagnosis; iii) a recorded serum urate level ≤300 µmol/L within 12 months of index diagnosis; and iv) treat-to-target urate monitoring, which we defined as two or more serum urate levels performed within 12 months of index diagnosis and/or one or more urate levels ≤300 µmol/L within the same time period (i.e. representing a minimum threshold for treat-to-target monitoring). Attainment of these outcomes within 24 months of the index gout diagnosis date were also reported as secondary outcome measures for people with at least 24 months of follow-up with a CPRD practice after diagnosis.

Predictor variables were selected *a priori* on the basis of whether they were felt to be important potential confounders of outcome measures, as follows: age at gout diagnosis; sex; year of gout diagnosis; country within the United Kingdom where patients’ registered primary care practices were located (England, Wales, Scotland or Northern Ireland); comorbidities (CKD stages 3-5, hypertension, diabetes mellitus, IHD, heart failure, previous stroke or transient ischaemic attack (TIA), and obesity); current or previous history of urolithiasis; smoking status (current/previous smoker vs. never smoker); alcohol excess; and diuretic therapy at gout diagnosis. Definitions of comorbidities and included Read codes are available in the supplementary appendix.

### Statistical analysis

Baseline characteristics were tabulated and described without inferential statistics. Attainment of outcome measures by year of gout diagnosis were described graphically using two-way plots.

Interrupted time-series analyses (ITSA) were used to estimate the effect of the introduction of updated BSR and EULAR gout management guidelines (published in June 2017 and July 2016, respectively) on: i) the prescription of ULT, and ii) target urate attainment within 12 months of index gout diagnosis. Monthly averages of these outcomes were compared in the periods before and after the introduction of the updated guidelines using single-group ITSA. Autocorrelation between observation periods was accounted for using a Prais-Winsten approach, whereby the generalised least-squares method is used to estimate parameters in a regression model, in which standard errors are assumed to follow a first-order autoregressive process.^15^ Robust standard errors were used to allow for practice-level clustering.

Logistic regression was used to estimate the strength of associations between predictor variables and outcomes measures. Robust standard errors were estimated to account for clustering of patients within practices. Age and sex-adjusted models and fully-adjusted models (adjusted for all predictor variables, including year of gout diagnosis) were presented with odds ratios and 95% confidence intervals.

Cox proportional hazards models with robust standard errors were used to describe associations between predictor variables and the time to initiation of ULT following new gout diagnoses (single failure models). Age and sex-adjusted models and fully-adjusted models (adjusted for all predictor variables, including year of gout diagnosis) were presented with hazard ratios and 95% confidence intervals. Assumptions regarding proportional hazards were tested graphically using Nelson-Aalen and log-log plots.

Statistical analyses were performed in Stata version 17.1.

### Study approval and ethics

The study protocol was approved by the CPRD Research Data Governance committee (approval number: 21_000680). No further ethical approval was required.

### Role of the funding source

The funder had no role in the study design; in the collection, analysis, and interpretation of data; in the writing of the report; or in the decision to submit the paper for publication.

### Patient and public involvement

Patients were closely involved in the design of this study. Interviews were conducted with patients with gout to identify potentially important outcomes and to highlight areas of sub-optimal care. Specifically, patients reported infrequent ULT initiation, titration and urate monitoring after diagnosis, which formed primary outcome measures for our analyses. Going forward, patients, clinicians and members of the public will be invited to attend public engagement events to review the study findings and discuss follow-up projects, with the aim of improving the quality of care for people with gout.

## Results

### Baseline demographics and comorbidities

Within the cohort, 129,972 people from 905 practices had new diagnoses of gout between January 2004 and October 2020. The mean and median durations of follow-up were 6.3 years and 5.4 years, respectively. The mean age of patients at diagnosis was 62 years; 72.8% were male. 53.2% of patients were registered with a practice in England; 21.7% in Wales; 20.1% in Scotland; and 5.0% in Northern Ireland. Patient demographics, comorbidities and diuretic use at gout diagnosis are summarised in Table 1. The number of patients with newly-diagnosed gout, separated by year of diagnosis, is shown in Supplementary Table S1.

**Table 1:** Baseline demographics, comorbidities and diuretic use in people newly diagnosed with gout, separated by sex. Data are presented as mean (standard deviation) for continuous measures, and n (%) for categorical measures. CKD: chronic kidney disease; TIA: transient ischaemic attack. Baseline serum urate levels were available for 80,054 patients (male patients: *n* = 56,963; female patients: n=23,091).

	Total	Male	Female
	*N* = 129,972	*N* = 94,610	*N* = 35,362
Age at diagnosis, years	62 (15)	59 (15)	69 (14)
Country:			
England	69,129 (53.2%)	50,897 (53.8%)	18,232 (51.6%)
Wales	28,180 (21.7%)	20,387 (21.5%)	7793 (22.0%)
Scotland	26,154 (20.1%)	18,706 (19.8%)	7448 (21.1%)
Northern Ireland	6509 (5.0%)	4620 (4.9%)	1889 (5.3%)
Number of comorbidities at diagnosis	1.5 (1.4)	1.3 (1.3)	2.0 (1.4)
CKD stages 3-5			
No	97,103 (74.7%)	76,665 (81.0%)	20,438 (57.8%)
Yes	32,869 (25.3%)	17,945 (19.0%)	14,924 (42.2%)
Hypertension			
No	68,106 (52.4%)	55,387 (58.5%)	12,719 (36.0%)
Yes	61,866 (47.6%)	39,223 (41.5%)	22,643 (64.0%)
Diabetes mellitus			
No	114,309 (87.9%)	85,144 (90.0%)	29,165 (82.5%)
Yes	15,663 (12.1%)	9466 (10.0%)	6197 (17.5%)
Ischaemic heart disease			
No	109,817 (84.5%)	80,348 (84.9%)	29,469 (83.3%)
Yes	20,155 (15.5%)	14,262 (15.1%)	5893 (16.7%)
Heart failure			
No	121,084 (93.2%)	88,836 (93.9%)	32,248 (91.2%)
Yes	8888 (6.8%)	5774 (6.1%)	3114 (8.8%)
Previous stroke or TIA			
No	122,200 (94.0%)	89,570 (94.7%)	32,630 (92.3%)
Yes	7772 (6.0%)	5040 (5.3%)	2732 (7.7%)
Obesity			
No	79,364 (61.1%)	60,044 (63.5%)	19,320 (54.6%)
Yes	50,608 (38.9%)	34,566 (36.5%)	16,042 (45.4%)
Urolithiasis			
No	127,259 (97.9%)	92,432 (97.7%)	34,827 (98.5%)
Yes	2713 (2.1%)	2178 (2.3%)	535 (1.5%)
Current/ex-smoker			
No	48,550 (37.4%)	34,953 (36.9%)	13,597 (38.5%)
Yes	81,422 (62.6%)	59,657 (63.1%)	21,765 (61.5%)
Alcohol excess			
No	121,975 (93.8%)	87,823 (92.8%)	34,152 (96.6%)
Yes	7997 (6.2%)	6787 (7.2%)	1210 (3.4%)
Diuretic therapy			
No	82,986 (63.8%)	67,916 (71.8%)	15,070 (42.6%)
Yes	46,986 (36.2%)	26,694 (28.2%)	20,292 (57.4%)
Baseline serum urate level, µmol/L	472	480	452

At diagnosis, 72.5% of patients had one or more of the following comorbidities: hypertension (47.6%); CKD stages 3-5 (25.3%); diabetes mellitus (12.1%); IHD (15.5%); heart failure (6.8%); previous stroke or TIA (6.0%); and/or obesity (38.9%). 36.2% of patients were receiving diuretic therapy at diagnosis, and 2.1% of patients had a current or previous history of urolithiasis. 61.6% of patients had a baseline serum urate performed, with a mean level of 472 µmol/L. Baseline urate levels were higher in male than female patients (480 µmol/L vs. 452 µmol/L, respectively) and in patients with a greater comorbidity burden at presentation (440 µmol/L vs. 612 µmol/L, respectively, in patients with no comorbidities vs. seven comorbidities at presentation).

### Prescription of ULT after diagnosis

**Figure 1:**
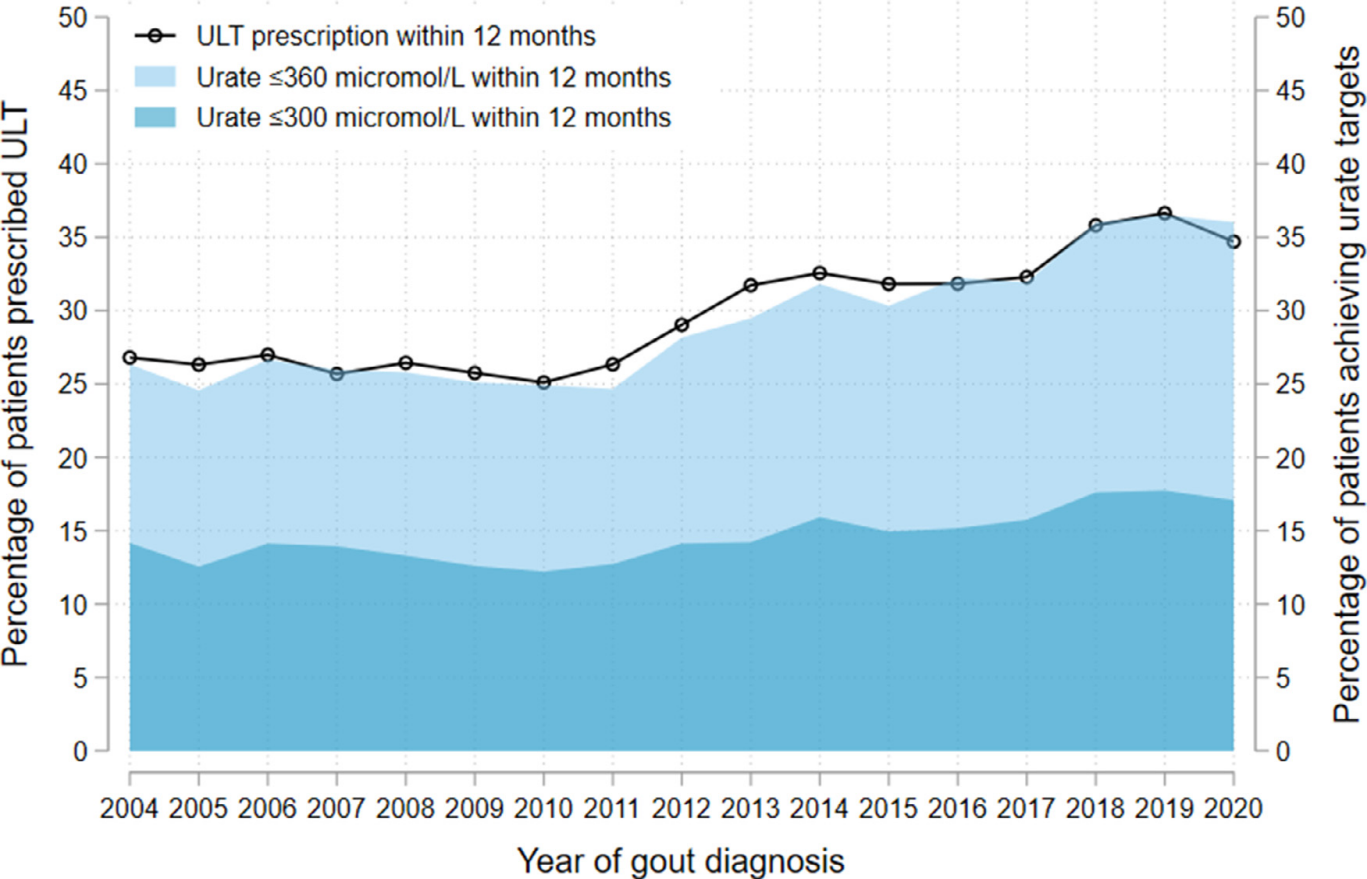
Proportion of patients newly diagnosed with gout (*n* = 129,972), separated by year of diagnosis, who: i) were initiated on urate-lowering therapy (ULT) within 12 months of diagnosis (black line); or ii) had a serum urate performed (*n* = 65,127) and attained a level ≤360 µmol/L (light blue) or ≤300 µmol/L (dark blue) within 12 months of diagnosis. The total number of patients, separated by year of diagnosis, sex, and whether a serum urate was performed is provided in Supplementary Table S1.

Overall, 37,529 (28.9%) of 129,972 people with newly-diagnosed gout received prescriptions for ULT within 12 months of diagnosis. The proportion of people initiated on ULT within 12 months of diagnosis improved modestly over the study period, from 26.8% for those diagnosed in 2004 to 36.6% in 2019, decreasing slightly to 34.7% for people diagnosed in 2020 (Figure 1 and Supplementary Figure S1).

**Figure 2:**
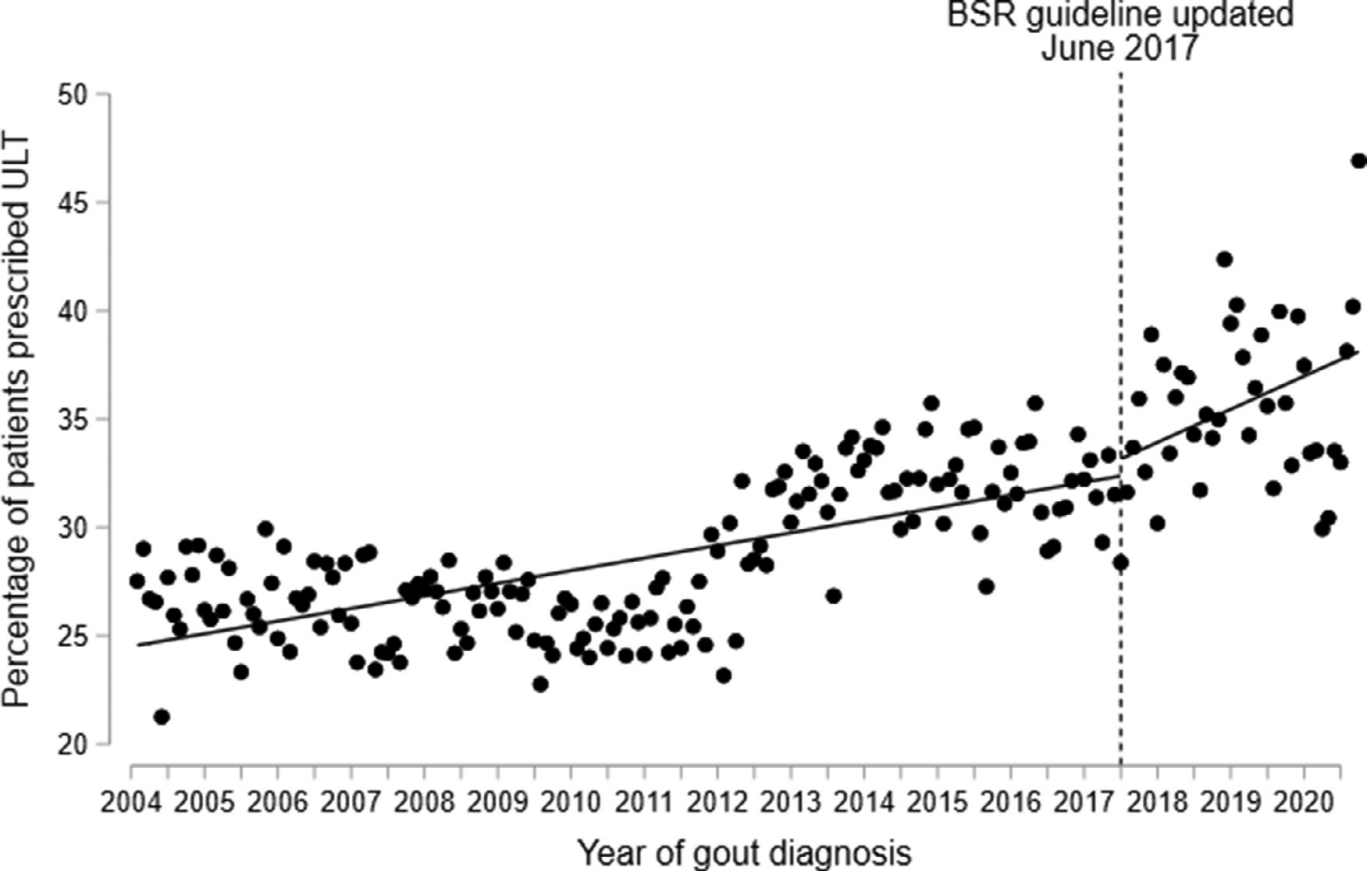
Percentage of newly-diagnosed gout patients (*n* = 129,972) who were prescribed urate-lowering therapy (ULT) within 12 months of diagnosis, comparing trends before and after the introduction of the updated BSR gout management guideline (published in June 2017). Trends were assessed using interrupted time-series analysis, with single time point dots representing monthly average percentages of ULT initiation.

We estimated the effect of publication of updated BSR and EULAR gout management guidelines (in June 2017 and July 2016, respectively) on the initiation of ULT, using ITSA models. The trend in ULT initiation after publication of the BSR guideline was not significantly different to prior to publication (rate of improvement post-guideline: 1.53% per year; pre-guideline: 0.58% per year; difference: 0.95% per year: 95% CI -1.13 to 3.02, *p* = 0.37) (Figure 2). Similarly, no statistically significant differences in ULT initiation were observed after publication of the EULAR guideline (rate of improvement post-guideline: 1.61% per year; pre-guideline: 0.58% per year; difference: 1.03% per year: 95% CI -0.14 to 2.21, *p* = 0.09) (Supplementary Figure S2). As sensitivity analyses, ITSA were performed with an additional cut point in January 2011 (i.e. before an apparent improvement in ULT initiation between 2011 and 2014); this demonstrated improvements in ULT initiation after 2011, relative to before 2011, but showed no significant changes after the publication of BSR or EULAR guidelines (Supplementary Figures S3 and S4).

Of first ULT prescriptions, 37,293 (99.4%) were for allopurinol, while 222 (0.6%) were for febuxostat. The proportion of first ULT prescriptions for medications other than allopurinol increased from <0.15% prior to 2011, to 1.7% in 2017, before decreasing slightly to 1.1% in 2019 (Supplementary Figure S5). There were no first ULT prescriptions for medications other than allopurinol in 2020.

### Target serum urate attainment and monitoring

65,127 (50.1%) of 129,972 people with newly-diagnosed gout had at least one serum urate level performed within 12 months of diagnosis, of whom 9304 (14.3%) attained a serum urate level ≤300 µmol/L and 18,523 (28.4%) attained a level ≤360 µmol/L. Target urate attainment increased modestly over the study period, from 14.2% in 2004 to 17.7% in 2019 and 17.1% in 2020 (for ≤300 µmol/L), and from 26.3% in 2004 to 36.5% in 2019 and 36.0% in 2020 (for ≤360 µmol/L) (Figure 1). In ITSA models, trends in the attainment of urate targets after publication of updated EULAR and BSR guidelines were not significantly different to prior to the publication of these guidelines (Supplementary Figures S6-S9).

24,593 (18.9%) of 129,972 patients received treat-to-target serum urate monitoring, which we defined as two or more serum urate levels performed within 12 months of diagnosis and/or one or more serum urate levels ≤300 µmol/L within the same period. Treat-to-target monitoring increased from 15.9% of patients in 2004 to 28.2% of patients in 2018, before decreasing to 22.4% in 2020 (Supplementary Figure S10).

Trends in the attainment of serum urate targets, initiation of ULT, and treat-to-target urate monitoring within 24 months of gout diagnosis were comparable to those observed within 12 months of diagnosis, as shown in Supplementary Figures S11 and S12. Temporal trends in ULT initiation and urate target attainment, comparing male and female patients, are shown in Supplementary Figure S13.

### Predictors of ULT prescription and target urate attainment

Univariable and multivariable logistic regression was performed to analyse predictors of ULT prescription (Table 2) and attainment of serum urate levels ≤300 µmol/L (Table 3) or ≤360 µmol/L (Supplementary Table S2) within 12 months of gout diagnosis.

**Table 2:** Predictors of ULT initiation within 12 months of gout diagnosis. Univariable logistic regression outputs are shown (adjusted for age at diagnosis and sex), in addition to multivariable logistic regression outputs (with adjustment for all predictor variables, including year of diagnosis). Robust standard errors were estimated to account for clustering of patients within practices. CKD: chronic kidney disease; TIA: transient ischaemic attack.

Variables	Odds ratio (univariable)	95% CI	*p*-value	Odds ratio (multivariable)	95% CI	*p*-value
Age at diagnosis (per 10-year increase)	1.03	(1.02 - 1.04)	<0.001	0.90	(0.89 - 0.91)	<0.001
Female sex	1.06	(1.03 - 1.09)	<0.001	0.90	(0.88 - 0.93)	<0.001
Year of gout diagnosis	1.03	(1.03 - 1.04)	<0.001	1.03	(1.02 - 1.03)	<0.001
Country:						
England	Reference			Reference		
Wales	1.00	(0.92 - 1.10)	0.91	0.93	(0.85 - 1.01)	0.09
Scotland	1.87	(1.71 - 2.05)	<0.001	1.65	(1.50 - 1.81)	<0.001
Northern Ireland	1.84	(1.58 - 2.15)	<0.001	1.65	(1.42 - 1.92)	<0.001
CKD stages 3-5	1.96	(1.89 - 2.02)	<0.001	1.61	(1.55 - 1.67)	<0.001
Hypertension	1.37	(1.33 - 1.41)	<0.001	1.06	(1.03 - 1.10)	<0.001
Diabetes mellitus	1.31	(1.26 - 1.36)	<0.001	1.01	(0.98 - 1.05)	0.49
Ischaemic heart disease	1.34	(1.30 - 1.39)	<0.001	1.08	(1.04 - 1.12)	<0.001
Heart failure	2.07	(1.97 – 2.18)	<0.001	1.56	(1.48 - 1.64)	<0.001
Previous stroke or TIA	1.14	(1.09 - 1.20)	<0.001	0.98	(0.93 - 1.03)	0.34
Urolithiasis	1.20	(1.10 - 1.30)	<0.001	1.08	(0.99 - 1.18)	0.07
Obesity	1.46	(1.42 - 1.49)	<0.001	1.32	(1.29 - 1.36)	<0.001
Current/ex-smoker	1.04	(1.01 - 1.07)	0.01	0.98	(0.95 - 1.01)	0.19
Alcohol excess	1.27	(1.20 - 1.34)	<0.001	1.10	(1.04 - 1.17)	<0.001
Diuretic therapy	1.81	(1.76 - 1.87)	<0.001	1.49	(1.44 - 1.55)	<0.001

**Table 3:** Predictors of attainment of serum urate levels ≤300 µmol/L within 12 months of gout diagnosis. Univariable logistic regression outputs are shown (adjusted for age at diagnosis and sex), in addition to multivariable logistic regression outputs (with adjustment for all predictor variables, including year of diagnosis). Robust standard errors were estimated to account for clustering of patients within practices. CKD: chronic kidney disease; TIA: transient ischaemic attack.

Variables	Odds ratio (univariable)	95% CI	*p*-value	Odds ratio (multivariable)	95% CI	*p*-value
Age at diagnosis (per 10-year increase)	0.91	(0.89 - 0.93)	<0.001	1.05	(1.03 - 1.07)	<0.001
Female sex	3.99	(3.77 - 4.23)	<0.001	5.18	(4.86 - 5.53)	<0.001
Year of gout diagnosis	1.02	(1.02 - 1.03)	<0.001	1.02	(1.01 - 1.02)	<0.001
Country:						
England	Reference			Reference		
Wales	1.02	(0.95 - 1.10)	0.60	1.00	(0.92 - 1.08)	0.95
Scotland	1.20	(1.11 - 1.30)	<0.001	1.24	(1.14 - 1.35)	<0.001
Northern Ireland	1.52	(1.32 - 1.75)	<0.001	1.59	(1.37 - 1.85)	<0.001
CKD stages 3-5	0.47	(0.44 - 0.51)	<0.001	0.51	(0.48 - 0.55)	<0.001
Hypertension	0.60	(0.57 - 0.64)	<0.001	0.84	(0.79 - 0.89)	<0.001
Diabetes mellitus	0.89	(0.82 - 0.95)	<0.001	1.19	(1.10 - 1.28)	<0.001
Ischaemic heart disease	0.84	(0.78 - 0.90)	<0.001	1.00	(0.93 - 1.08)	0.99
Heart failure	0.62	(0.56 - 0.69)	<0.001	0.85	(0.76 - 0.95)	<0.001
Previous stroke or TIA	0.90	(0.81 - 1.00)	0.04	1.02	(0.92 - 1.13)	0.66
Urolithiasis	1.19	(1.02 - 1.38)	0.02	1.25	(1.07 - 1.46)	<0.001
Obesity	0.58	(0.55 - 0.61)	<0.001	0.61	(0.58 - 0.65)	<0.001
Current/ex-smoker	1.14	(1.08 - 1.20)	<0.001	1.18	(1.12 - 1.24)	<0.001
Alcohol excess	1.19	(1.08 - 1.32)	<0.001	1.05	(0.95 - 1.17)	0.32
Diuretic therapy	0.47	(0.44 - 0.50)	<0.001	0.61	(0.57 - 0.66)	<0.001

People with gout and CKD stages 3-5 at baseline were more likely to be prescribed ULT within 12 months of diagnosis than patients without CKD (adjusted mean difference 9.83%, adjusted OR 1.61, 95% CI 1.55 to 1.67, *p* < 0.001); however, they were less likely to achieve serum urate levels ≤300 µmol/L within 12 months of diagnosis (adjusted mean difference -6.74%, adjusted OR 0.51, 95% CI 0.48 to 0.55, *p* < 0.001).

Similar findings of increased ULT prescription but decreased urate target attainment were observed for people with the following comorbidities at baseline: heart failure (ULT prescription: adjusted mean difference 9.32%, adjusted OR 1.56, 95% CI 1.48 to 1.64, *p* < 0.001; target attainment: adjusted mean difference -1.72%, adjusted OR 0.85, 95% CI 0.76 to 0.95, *p* < 0.001); hypertension (ULT prescription: adjusted mean difference 1.22%, adjusted OR 1.06, 95% CI 1.03 to 1.10, *p* < 0.001; target attainment: adjusted mean difference -1.95%, adjusted OR 0.84, 95% CI 0.79 to 0.89, *p* < 0.001); obesity (ULT prescription: adjusted mean difference 5.53%, adjusted OR 1.32, 95% CI 1.29 to 1.36, *p* < 0.001; target attainment: adjusted mean difference -5.29%, adjusted OR 0.61, 95% CI 0.58 to 0.65, *p* < 0.001); and in patients receiving diuretic therapy at baseline (ULT prescription: adjusted mean difference 8.03%, adjusted OR 1.49, 95% CI 1.44 to 1.55, *p* < 0.001; target attainment: adjusted mean difference -5.27%, adjusted OR 0.61, 95% CI 0.57 to 0.66, *p* < 0.001). Comparable findings were observed for the attainment of target urate levels ≤360 µmol/L within 12 months of diagnosis (Supplementary Table S2).

**Figure 3:**
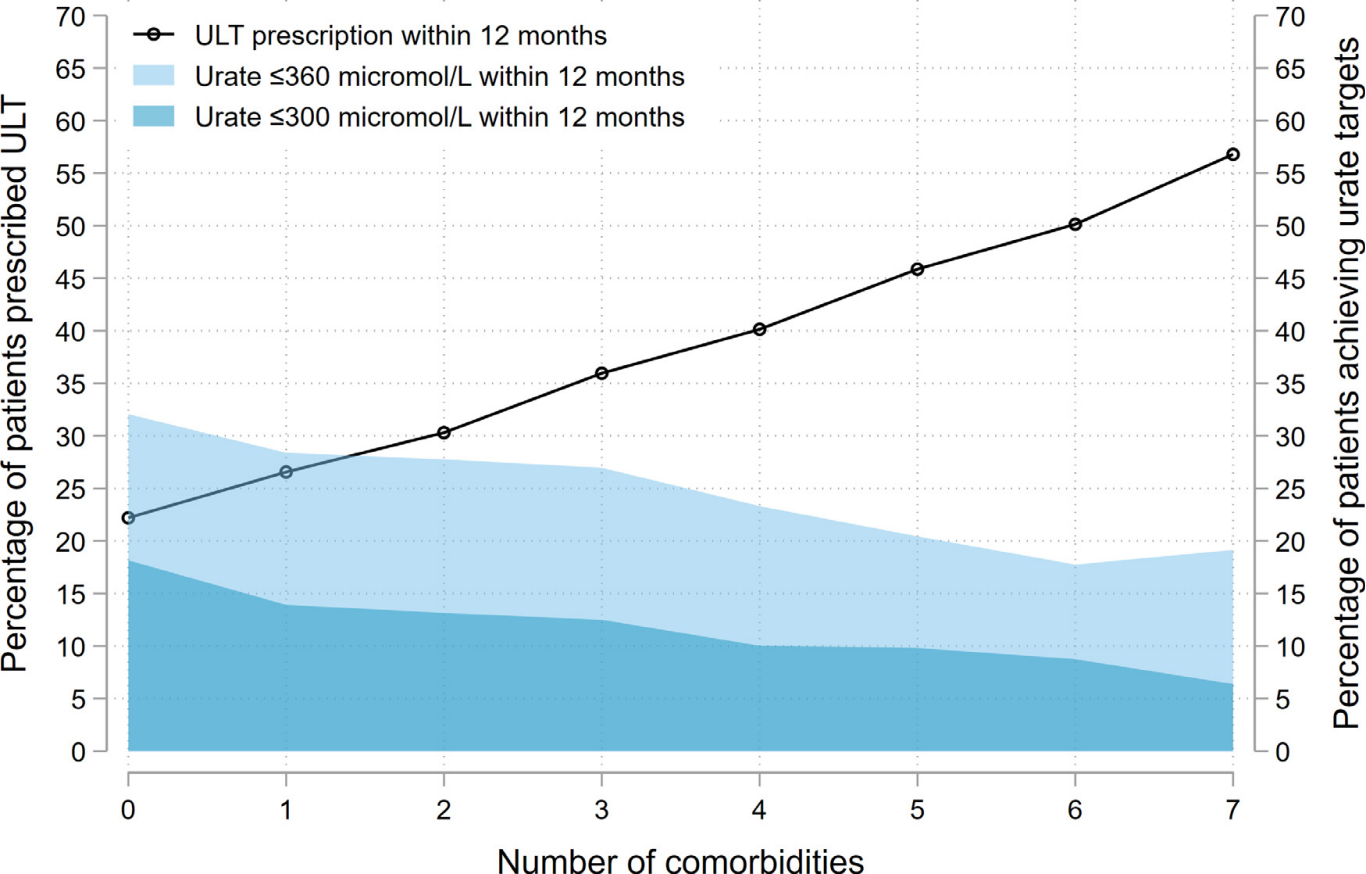
Impact of the number of comorbidities present at diagnosis on the proportion of newly-diagnosed gout patients (*n* = 129,972) who: i) were initiated on urate-lowering therapy (ULT) within 12 months of diagnosis (black line); or ii) had a serum urate performed (*n* = 65,127) and attained a level ≤360 µmol/L (light blue) or ≤300 µmol/L (dark blue) within 12 months of diagnosis. Comorbidities assessed at baseline were chronic kidney disease stages 3-5, hypertension, diabetes mellitus, ischaemic heart disease, heart failure, previous stroke/transient ischaemic attack and obesity.

The associations of multimorbidity on ULT prescription and urate target attainment were additive: each additional comorbidity present at gout diagnosis increased the likelihood of ULT prescription, but decreased the likelihood of urate target attainment within 12 months of diagnosis (Figure 3). The effect of multimorbidity on ULT initiation and urate target attainment was more pronounced for female than male patients (Supplementary Figure S14).

Female patients were much more likely than male patients to attain urate levels ≤300 µmol/L (adjusted mean difference 23.0%, adjusted OR 5.18, 95% CI 4.86 to 5.53, *p* < 0.001) and ≤360 µmol/L (adjusted mean difference 27.2%, adjusted OR 3.68, 95% CI 3.50 to 3.87, *p* < 0.001). In unadjusted analyses, slightly more female than male patients were initiated on ULT within 12 months of diagnosis (30.2% vs. 28.4%, respectively). However, after adjusting for other predictors, female patients were slightly less likely to be initiated on ULT than male patients, with the same true of older patients relative to younger patients (Table 2); this was primarily due to interaction effects between age, sex, CKD and diuretic use at baseline.

Patients registered with practices in Scotland or Northern Ireland were more likely to be initiated on ULT (Table 2) and to achieve target urate levels (Table 3 and Supplementary Table S2) within 12 months of diagnosis, relative to patients registered with practices in England.

Survival modelling was performed to analyse predictors of the time to first ULT prescription following new gout diagnoses (Supplementary Table S3), with results highly comparable to those observed in univariable and multivariable logistic regression models (Table 2).

## Discussion

In this UK-wide study, we show that the initiation of ULT, monitoring and attainment of target urate levels following new gout diagnoses remain poor, with only marginal improvements in these outcomes between 2004 and 2020. Even after the introduction of updated British and European gout management guidelines, only one in three people with gout are prescribed ULT within 12 months of diagnosis, and only one in six achieve a urate ≤300 µmol/L. These findings are a stark warning about the quality and success of gout care.

A previous study of UK gout management showed that, in 2012, 27% of people with gout were prescribed ULT within 12 months of diagnosis.^1^ Studies in other countries, including the United States,^6^, ^7^, ^8^ Australia,^9^ New Zealand,^10^ Sweden,^12^ and Taiwan^11^ also reported sub-optimal ULT initiation and target attainment. In 2016 and 2017, respectively, EULAR and BSR gout management guidelines were updated to encourage the prescription of ULT, with titration of the dose of ULT until a target serum urate level is achieved.^3^^,^^13^ Despite this, in time-series analyses, we showed that trends in the prescription of ULT and the attainment of urate targets after publication of these guidelines were not significantly different to before publication. We also observed reductions in the prescription of ULT and in urate monitoring in people diagnosed in 2020, relative to 2019. This is likely, at least in part, to reflect reduced access to care as a consequence of the COVID-19 pandemic, which has been reported for other chronic diseases.^16^ The pandemic is also likely to have impacted on clinician uptake of new guidance. Given the relatively short timeframe between the publication of updated BSR and EULAR guidelines and the start of the pandemic, future analyses will provide more insight into the impact of the COVID-19 pandemic on gout care and guideline implementation.

Our findings suggest that, for there to be a step-change in the quality of gout care, implementation strategies are needed to complement guidelines and encourage the uptake of treat-to-target ULT by clinicians. The failure to adopt new guidance in primary care should not be seen as a failure of primary care itself, but rather as a systems failure. Patient and clinician education programmes are needed to disseminate key guidance and raise awareness about inequities in care. Enhanced modules within electronic patient records could automatically flag those in need of ULT initiation, titration, and monitoring. Financial incentives to encourage ULT prescription and target attainment could be explored, as has been done for other conditions (e.g. the Quality and Outcomes Framework in the UK). New models of care for people with gout may be needed: for example, engaging allied health professionals (e.g. nurses and pharmacists) from primary care or community pharmacies in ULT titration and monitoring, which has been shown to be effective.^5^^,^^17^^,^^18^ Point-of-care urate meters are also widely available, providing reliable estimates of urate levels to facilitate remote monitoring,^19^^,^^20^ while empowering patients to be in control of their condition.

Guidelines strongly advise initiation of ULT in people with gout who have risk factors that include CKD, hypertension, heart failure, and diuretic use.^3^^,^^13^ We found that patients with these risk factors were indeed more likely to be prescribed ULT within 12 months of diagnosis than patients without these risk factors; however, they were less likely to achieve target urate levels, leaving them at risk of ongoing flares and morbidity. The effects of multimorbidity on ULT prescription and urate target attainment were additive, suggesting that it is not only individual risk factors that influence whether ULT is titrated adequately, but also the comorbidity burden at diagnosis. Clear guidance is needed on the management of gout in the presence of comorbidities, to ensure that patients most at risk of poor outcomes receive adequate ULT titration. This is particularly true of CKD, in view of conflicting guidance on the maximum recommended doses of allopurinol in renal impairment.^3^^,^^13^^,^^21^ Conditions including cardiovascular disease, CKD and obesity have been shown to be associated with a greater urate burden.^22^^,^^23^ In our study, we showed that a greater comorbidity burden at diagnosis was associated with higher baseline serum urate levels, which may be contributing to the failure to adequately suppress urate levels in these patients. Although not specifically addressed in our study, it is also true that comorbidities, particularly renal impairment, influence clinicians’ willingness to dose-escalate ULT.^24^ Additionally, medications used to manage comorbidities, for example diuretics, impact upon the relative efficacy of ULT.^25^

Further research is needed to explore our finding that patients in Scotland and Northern Ireland are more likely to be prescribed ULT and achieve urate targets than patients in England or Wales. In 2012, Scotland and Northern Ireland had the lowest prevalence of gout in the UK,^1^ suggesting that the improved outcomes observed in these countries are not due to increased clinician exposure to the underlying condition. The differences in gout care are more likely to reflect better attainment of care quality indicators more generally in Scotland and Northern Ireland, relative to the rest of the UK: reports published by the National Audit Office and The Heath Foundation showed that practices in Scotland and Northern Ireland achieved the highest quality indicator scores in the UK.^26^^,^^27^

Our finding that female patients were five times more likely than male patients to obtain a target urate level ≤300 µmol/L also warrants further investigation. This finding was not explained by differences in ULT initiation, with female patients being relatively less likely to be initiated on ULT than male patients after adjustment for other covariates. The differences may relate to lower serum urate levels at baseline in female than male patients, contributing to easier attainment of target urate levels. Additionally, there may be differences in how male and female patients respond to ULT; in medication adherence; and other aspects of given care, which should be explored in future studies.

The strengths of our study include its population-level data coverage, large sample size, high quality and comprehensive data source,^28^^,^^29^ and study period of greater than 15 years. In addition to analysing trends in ULT prescription, we also investigated trends in urate target attainment and monitoring, as well as predictors of these outcomes. Our statistical models accounted for multiple potential confounders, including year of diagnosis, recognising that clinical practice evolves with changing guidance over time.

Our study had limitations. The study was performed on a UK-based primary care cohort and, although comparable results have been reported in many countries,^30^ the findings should not be assumed to be generalisable to other countries or settings. For example, the American College of Physicians recommended a “treat-to-avoid-symptoms” approach rather than a “treat-to-urate-target” approach,^31^ while the American College of Rheumatology conditionally recommended against ULT initiation after first gout flares in the absence of specific risk factors.^32^ While BSR guidance recommends offering ULT to all patients with gout and EULAR guidance recommends considering and discussing ULT with gout patients from the first presentation, our analyses did not account for cases where ULT was offered to patients but declined or investigate prescription trends in patients with vs. without definite indications for ULT (e.g. urolithiasis).

The case definition for gout used in our analyses was based upon clinical codes entered by general practitioners, rather than classification criteria or urate crystal identification. As such, there is the potential for diagnostic misclassification inherent to analyses of clinically-coded data without case verification. Similarly, there is the potential for misclassification with clinical coding of comorbidities and missing data. Previous studies have, however, demonstrated the high validity of diagnostic coding in CPRD, including gout coding.^28^^,^^29^ Our analyses did not account for the potential impact of gout flares on urate levels (i.e. lowering of urate levels during flares) or the impact of symptoms on medication adherence and/or attendance for blood tests, both of which are important areas for future study. Whilst we adjusted for multiple predictor variables in our models, the potential for unmeasured confounding must be considered when interpreting associations; for example, we did not have access data on ethnicity or socioeconomic indices. Although practice region was adjusted for in our models, one must consider the potential impact of changes in the regional composition of CPRD on temporal trends in ULT prescription and target attainment, with reducing numbers of CPRD GOLD-contributing practices from England since 2004 and increasing relative proportions of Welsh, Scottish and Northern Irish-contributing practices.

In conclusion, only a minority of people with gout in the UK are initiated on ULT or attain target urate levels within 12 months of diagnosis. This is despite the introduction of guidelines that lowered the threshold for ULT initiation and recommended titration of ULT dosing until target urate levels are achieved. If the evidence-practice gap in gout management is to be bridged successfully, implementation strategies that incorporate multiple complementary approaches are required, integrating primary and secondary care, and including education programmes and incentivisation.

## Contributors

MDR and JBG conceptualised the study. MDR, MCG, JBG, SN, AD, APC, AIR and BE contributed to the study design and methodology. MDR and JBG created the code lists for outcomes and predictor variables. MDR and MCG curated the data. MDR performed formal analyses in consultation with JBG, MCG, SN and AD. MDR, MBG, JBG, SN, AD, APC, AIR and BE contributed to interpretation of the results. MDR drafted the manuscript, with revision and review by all authors. All authors approved the final manuscript. The corresponding author directly accessed and verified the underlying data reported in the manuscript; all authors had full access to the data in the study and accept responsibility to submit for publication.

## Data Sharing Statement

The anonymised, coded data used in these analyses were provided by CPRD following approval by their Research Data Governance committee. The data are available on request from CPRD. Additional code lists used in these analyses are available on request from the corresponding authors.

## Declaration of interests

All authors have completed the ICMJE uniform disclosure form at http://www.icmje.org/disclosure-of-interest/ and declare: MDR has received honoraria from Lilly and Menarini, and support for attending meetings from Pfizer, Lilly, Janssen and UCB; JG has received honoraria from Abbvie, Biovitrum, BMS, Celgene, Chugai, Gilead, Janssen, Lilly, Novartis, Pfizer, Roche, Sanofi, Sobi and UCB; no other authors reported relationships or activities that could appear to have influenced the submitted work.

## Appendix Supplementary materials

Image, application 1

Image, application 2
