# Supplementary material for: Management of gout following 2016/2017 European (EULAR) and British (BSR) guidelines: An interrupted time-series analysis in the United Kingdom
Source: Lancet Reg Health Eur. 2022 May 25;18:100416. doi: 10.1016/j.lanepe.2022.100416 (PMC9257653; doi:10.1016/j.lanepe.2022.100416)
Supplement: Supplementary file 1 [file mmc1.docx]

**Captions for Supplementary Material**

Supplementary Figure S1. Initiation of ULT and attainment of urate targets within 12 months of gout diagnosis, including patient counts by year of diagnosis.

Supplementary Figure S2. Interrupted time-series analysis of ULT initiation, before and after the introduction of updated EULAR guidelines.

Supplementary Figure S3. Interrupted time-series sensitivity analysis, assessing ULT initiation with two cut points: January 2011 and after the introduction of updated BSR guidelines.

Supplementary Figure S4. Interrupted time-series sensitivity analysis, assessing ULT initiation with two cut points: January 2011 and after the introduction of updated EULAR guidelines.

Supplementary Figure S5. Initiation of ULT medications other than allopurinol, by year of diagnosis.

Supplementary Figure S6. Interrupted time-series analysis of attainment of urate levels ≤360 µmol/L, before and after the introduction of updated EULAR guidelines.

Supplementary Figure S7. Interrupted time-series analysis of attainment of urate levels ≤300 µmol/L, before and after the introduction of updated EULAR guidelines.

Supplementary Figure S8. Interrupted time-series analysis of attainment of urate levels ≤360 µmol/L, before and after the introduction of updated BSR guidelines.

Supplementary Figure S9. Interrupted time-series analysis of attainment of urate levels ≤300 µmol/L, before and after the introduction of updated BSR guidelines.

Supplementary Figure S10. Treat-to-target urate monitoring within 12 months of gout diagnosis, by year of diagnosis.

Supplementary Figure S11. Initiation of ULT and attainment of urate targets within 24 months of gout diagnosis, separated by year of diagnosis.

Supplementary Figure S12. Treat-to-target urate monitoring within 24 months of gout diagnosis, separated by year of diagnosis.

Supplementary Figure S13. Temporal trends in the initiation of ULT and attainment of urate targets within 12 months of diagnosis for male and female patients with gout.

Supplementary Figure S14. Impact of multimorbidity on ULT initiation and urate target attainment for male and female patients with gout.

Supplementary Table S1. Number of patients with newly-diagnosed gout, separated by sex, year of diagnosis and serum urate data availability.

Supplementary Table S2. Predictors of attainment of serum urate levels ≤360 µmol/L within 12 months of gout diagnosis.

Supplementary Table S3. Predictors of time to ULT initiation following new gout diagnoses, using Cox proportional hazards.

Read codes and definitions.
