# Supplementary material for: Management of gout following 2016/2017 European (EULAR) and British (BSR) guidelines: An interrupted time-series analysis in the United Kingdom
Source: Lancet Reg Health Eur. 2022 May 25;18:100416. doi: 10.1016/j.lanepe.2022.100416 (PMC9257653; doi:10.1016/j.lanepe.2022.100416)
Supplement: Supplementary file 2 [file mmc2.docx]

**Table of Contents**

[Supplementary Figure S1. Initiation of ULT and attainment of urate targets within 12 months of gout diagnosis, including patient counts by year of diagnosis. 2](#_Toc102936551)

[Supplementary Figure S2. Interrupted time-series analysis of ULT initiation, before and after the introduction of updated EULAR guidelines. 3](#_Toc102936552)

[Supplementary Figure S3. Interrupted time-series sensitivity analysis, assessing ULT initiation with two cut points: January 2011 and after the introduction of updated BSR guidelines. 4](#_Toc102936553)

[Supplementary Figure S4. Interrupted time-series sensitivity analysis, assessing ULT initiation with two cut points: January 2011 and after the introduction of updated EULAR guidelines. 5](#_Toc102936554)

[Supplementary Figure S5. Initiation of ULT medications other than allopurinol, by year of diagnosis 6](#_Toc102936555)

[Supplementary Figure S6. Interrupted time-series analysis of attainment of urate levels ≤360 µmol/L, before and after the introduction of updated EULAR guidelines. 7](#_Toc102936556)

[Supplementary Figure S7. Interrupted time-series analysis of attainment of urate levels ≤300 µmol/L, before and after the introduction of updated EULAR guidelines. 8](#_Toc102936557)

[Supplementary Figure S8. Interrupted time-series analysis of attainment of urate levels ≤360 µmol/L, before and after the introduction of updated BSR guidelines. 9](#_Toc102936558)

[Supplementary Figure S9. Interrupted time-series analysis of attainment of urate levels ≤300 µmol/L, before and after the introduction of updated BSR guidelines. 10](#_Toc102936559)

[Supplementary Figure S10. Treat-to-target urate monitoring within 12 months of gout diagnosis, by year of diagnosis 11](#_Toc102936560)

[Supplementary Figure S11. Initiation of ULT and attainment of urate targets within 24 months of gout diagnosis, separated by year of diagnosis. 12](#_Toc102936561)

[Supplementary Figure S12. Treat-to-target urate monitoring within 24 months of gout diagnosis, separated by year of diagnosis 13](#_Toc102936562)

[Supplementary Figure S13. Temporal trends in the initiation of ULT and attainment of urate targets within 12 months of diagnosis for male and female patients with gout. 14](#_Toc102936563)

[Supplementary Figure S14. Impact of multimorbidity on ULT initiation and urate target attainment for male and female patients with gout. 15](#_Toc102936564)

[Supplementary Table S1. Number of patients with newly-diagnosed gout, separated by sex, year of diagnosis and serum urate data availability. 16](#_Toc102936565)

[Supplementary Table S2. Predictors of attainment of serum urate levels ≤360 µmol/L within 12 months of gout diagnosis. 17](#_Toc102936566)

[Supplementary Table S3. Predictors of time to ULT initiation following new gout diagnoses, using Cox proportional hazards. 18](#_Toc102936567)

[Read codes and definitions 19](#_Toc102936568)

## **Supplementary Figure S1. Initiation of ULT and attainment of urate targets within 12 months of gout diagnosis, including patient counts by year of diagnosis.**


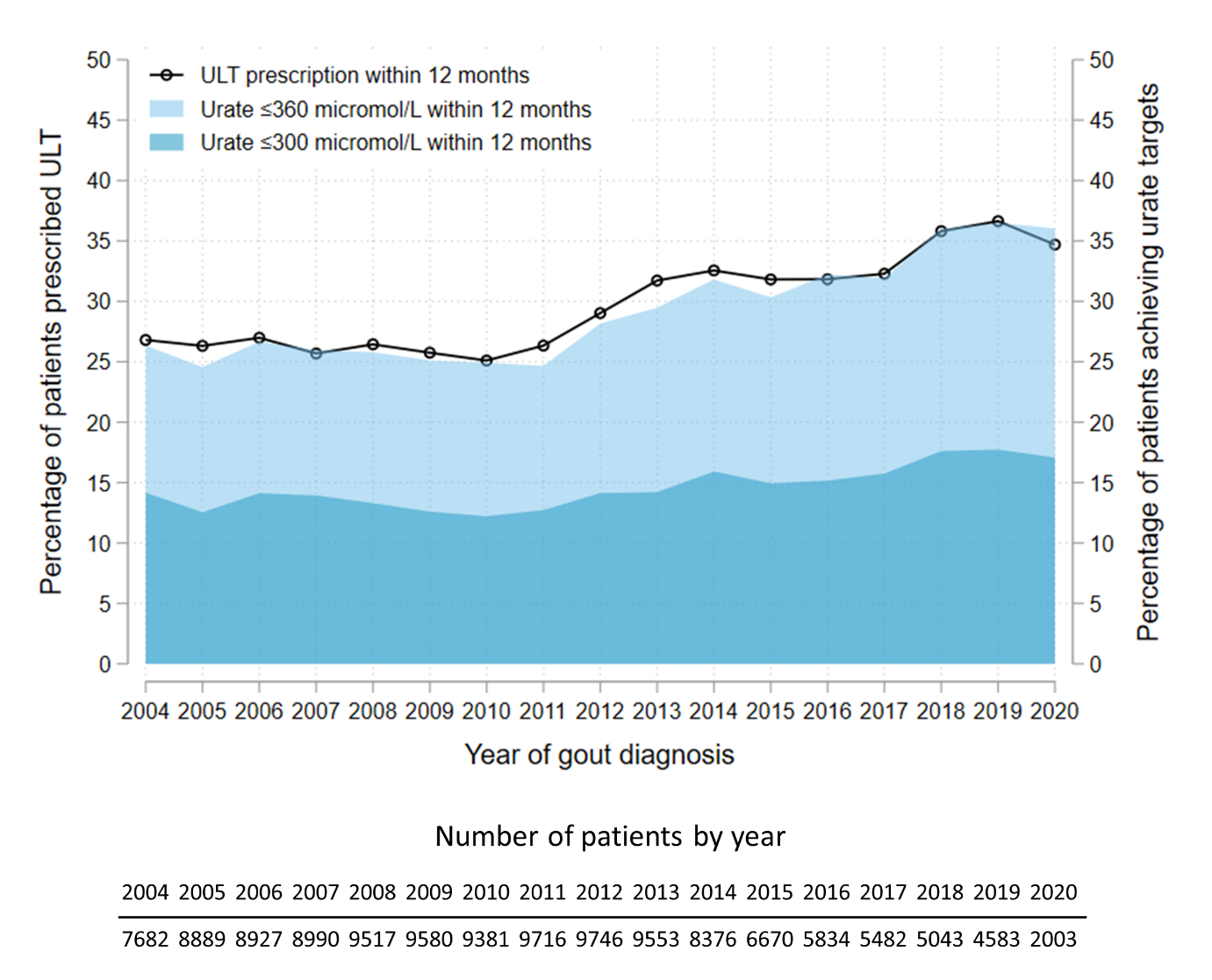


Proportion of patients newly diagnosed with gout (n=129,972), including patient counts by year of diagnosis, who: i) were initiated on urate-lowering therapy (ULT) within 12 months of diagnosis (black line); or ii) had a serum urate performed (n=65,127) and attained a level ≤360 µmol/L (light blue) or ≤300 µmol/L (dark blue) within 12 months of diagnosis. The total number of patients, separated by year of diagnosis, sex, and whether a serum urate was performed is provided in Supplementary Table S1.

## **Supplementary Figure S2. Interrupted time-series analysis of ULT initiation, before and after the introduction of updated EULAR guidelines.**


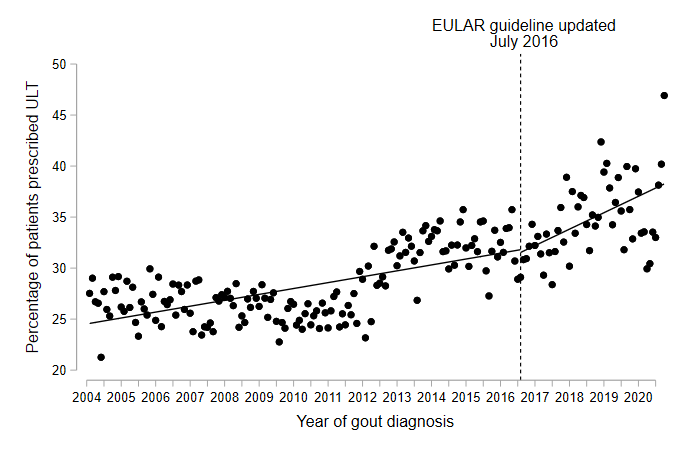


Percentage of patients newly diagnosed with gout (n=129,972) who were prescribed urate-lowering therapy (ULT) within 12 months of gout diagnosis, comparing trends before and after the introduction of the updated European Alliance of Associations for Rheumatology (EULAR) gout management guideline (published in July 2016). Trends were assessed using interrupted time-series analysis, with single time point dots representing monthly average percentages of ULT prescription rates.

## **Supplementary Figure S3. Interrupted time-series sensitivity analysis, assessing ULT initiation with two cut points: January 2011 and after the introduction of updated BSR guidelines.**

**
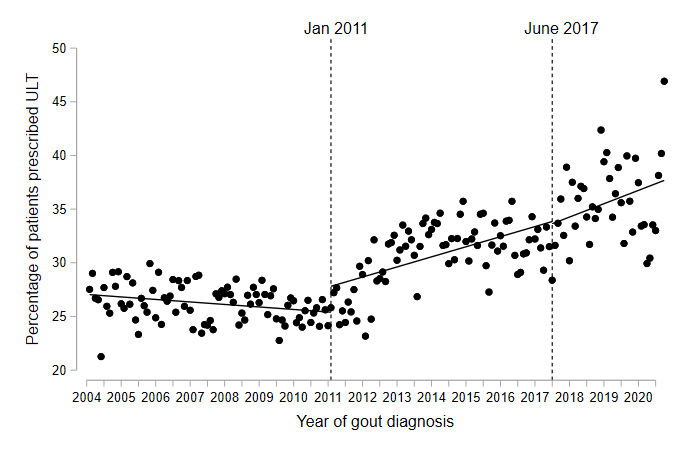
**

Percentage of patients newly diagnosed with gout (n=129,972) who were prescribed urate-lowering therapy (ULT) within 12 months of gout diagnosis, comparing trends before and after two time points: i) January 2011 (i.e. before an apparent period of improvement in ULT initiation between 2011 and 2014), and ii) after the introduction of updated British Society for Rheumatology (BSR) gout management guidelines in June 2017. Trends were assessed using interrupted time-series analysis, with single time point dots representing monthly average percentages of ULT prescription rates. Trend change after June 2017, relative to between Jan 2011 and June 2017: 0.30% improvement per year, 95% CI -1.50 to 2.10, p=0.74. Trend change between Jan 2011 and June 2017, relative to pre-2011: 1.17% improvement per year, 95% CI 0.71 to 1.63, p<0.001.

## **Supplementary Figure S4. Interrupted time-series sensitivity analysis, assessing ULT initiation with two cut points: January 2011 and after the introduction of updated EULAR guidelines.**


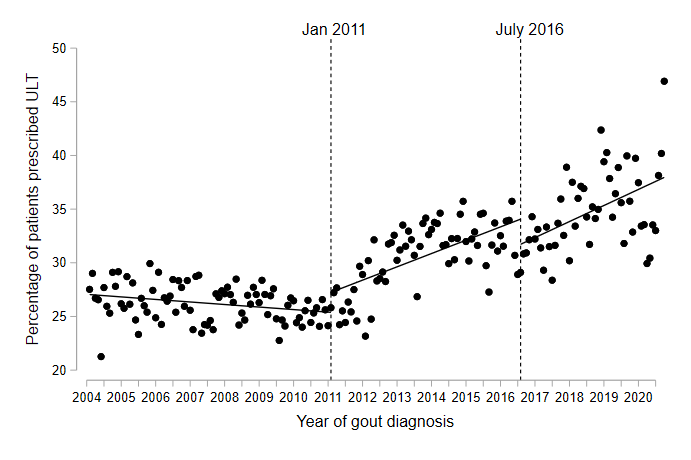


Percentage of patients newly diagnosed with gout (n=129,972) who were prescribed urate-lowering therapy (ULT) within 12 months of gout diagnosis, comparing trends before and after two time points: i) January 2011 (i.e. before an apparent period of improvement in ULT initiation between 2011 and 2014), and ii) after the introduction of updated EULAR gout management guidelines in July 2016. Trends were assessed using interrupted time-series analysis, with single time point dots representing monthly average percentages of ULT prescription rates. Trend change after July 2016, relative to between Jan 2011 and July 2016: 0.26% improvement per year, 95% CI -0.85 to 1.36, p=0.65. Trend change between Jan 2011 and July 2016, relative to pre-2011: 1.48% improvement per year, 95% CI 0.94 to 2.02, p<0.001.

## **Supplementary Figure S5. Initiation of ULT medications other than allopurinol, by year of diagnosis**


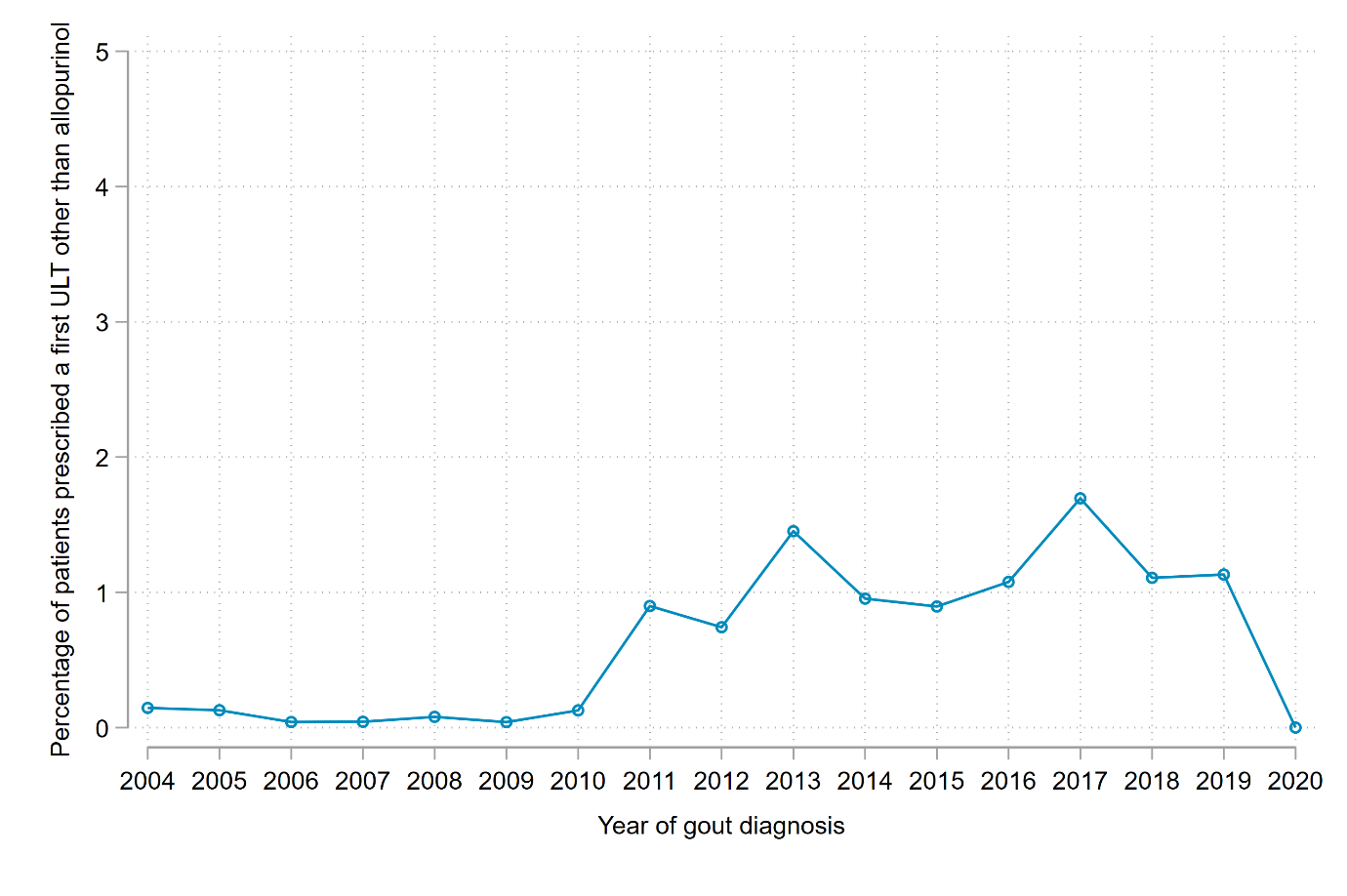


Percentage of patients newly diagnosed with gout (n=129,972) who received first ULT prescriptions for medications other than allopurinol within 12 months of diagnosis, separated by year of diagnosis.

## **Supplementary Figure S6. Interrupted time-series analysis of attainment of urate levels ≤360 µmol/L, before and after the introduction of updated EULAR guidelines.**

**
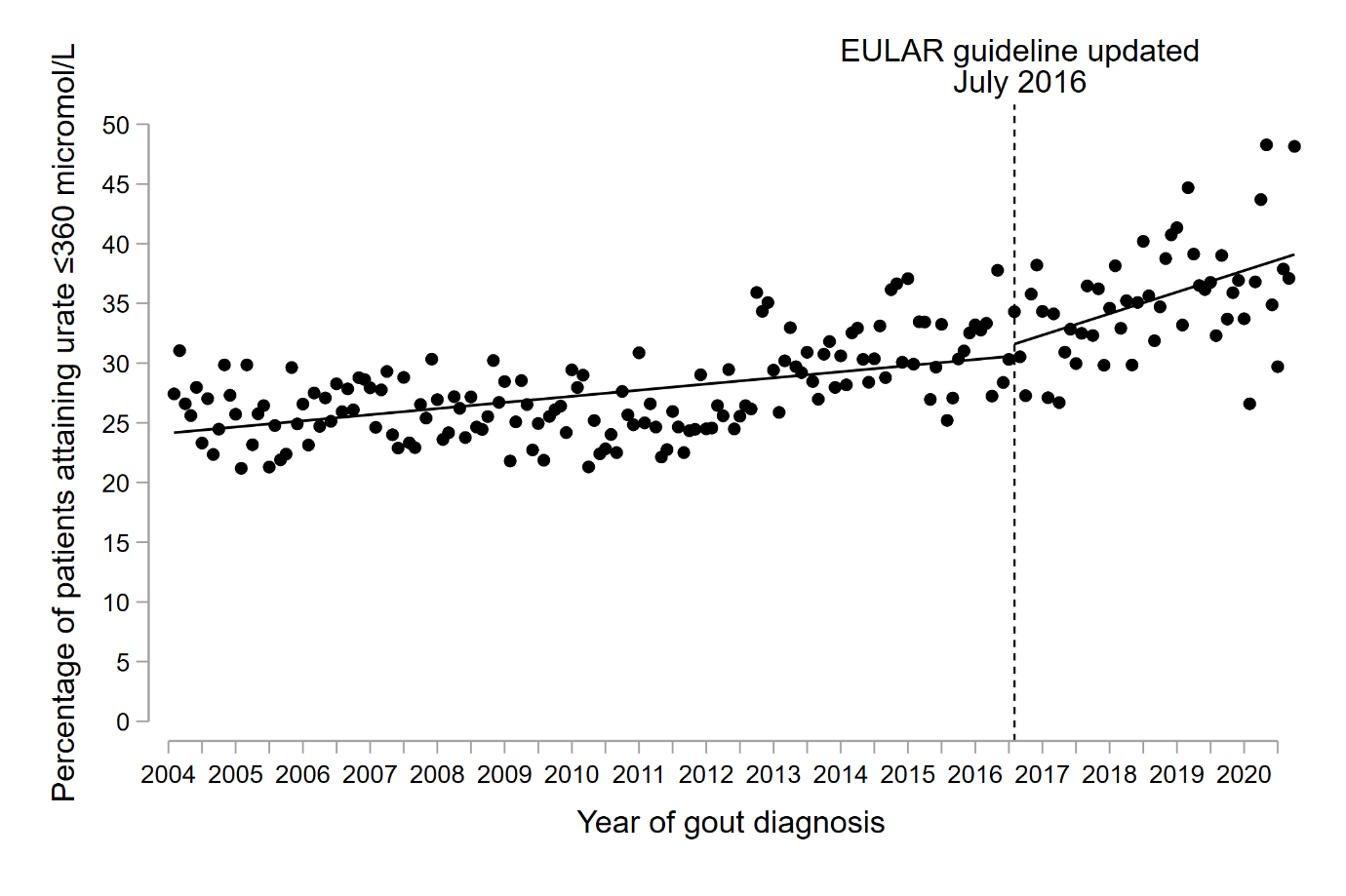
**

Percentage of patients newly diagnosed with gout who had a serum urate level performed within 12 months of diagnosis (n=65,127) and attained a urate ≤360 µmol/L, comparing trends before and after the introduction of the updated EULAR gout management guideline (published in July 2016). Trends were assessed using interrupted time-series analysis, with single time point dots representing monthly average percentages of urate target attainment rates. Rate of improvement post-guideline: 1.80% per year; pre-guideline: 0.51% per year; difference 1.28% per year, 95% CI -0.08 to 2.66, p=0.07.

## **Supplementary Figure S7. Interrupted time-series analysis of attainment of urate levels ≤300 µmol/L, before and after the introduction of updated EULAR guidelines.**


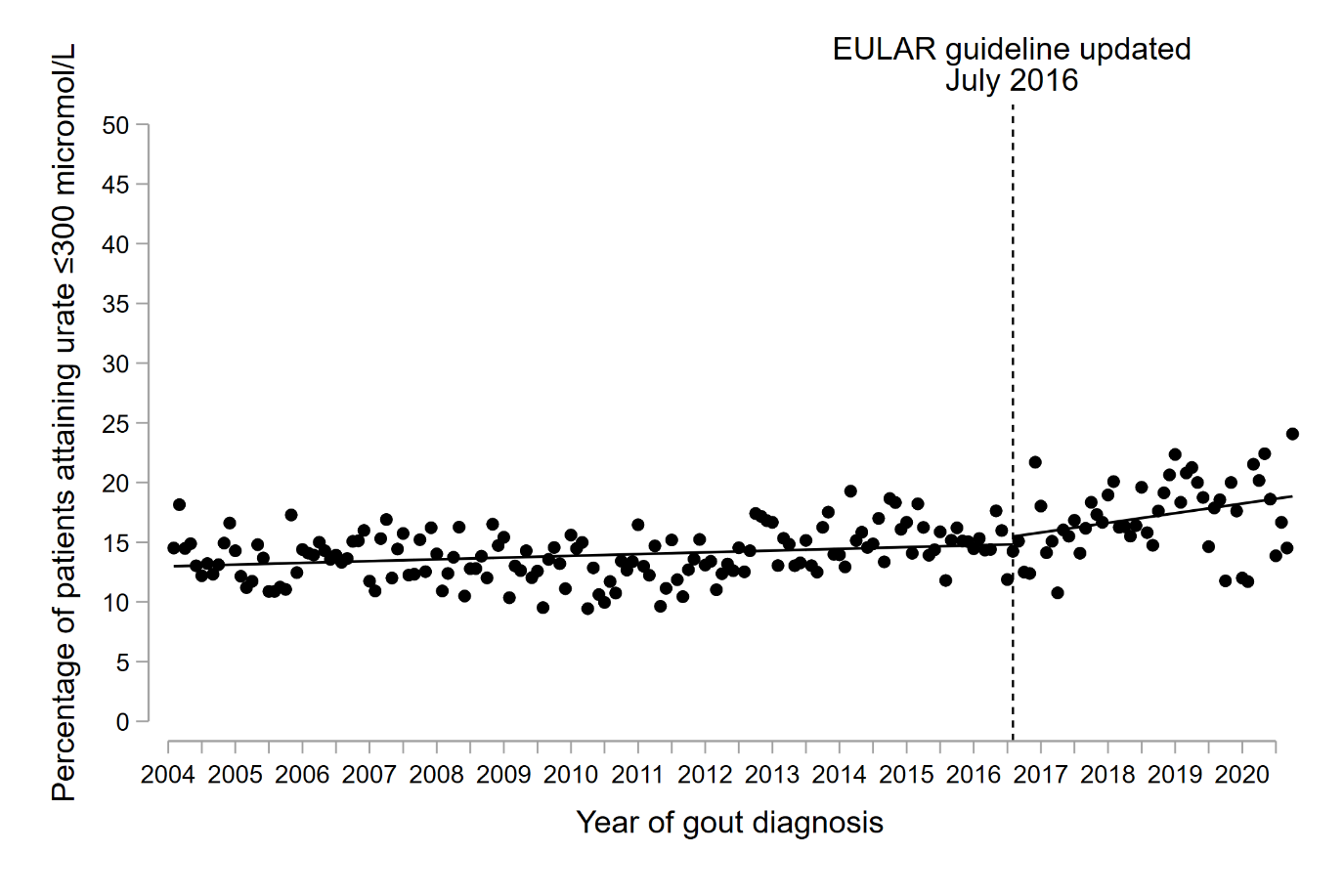


Percentage of patients newly diagnosed with gout who had a serum urate level performed within 12 months of diagnosis (n=65,127) and attained a urate ≤300 µmol/L, comparing trends before and after the introduction of the updated EULAR gout management guideline (published in July 2016). Trends were assessed using interrupted time-series analysis, with single time point dots representing monthly average percentages of urate target attainment rates. Rate of improvement post-guideline: 0.81% per year; pre-guideline: 0.15% per year; difference 0.66% per year, 95% CI -0.28 to 1.60, p=0.17.

## **Supplementary Figure S8. Interrupted time-series analysis of attainment of urate levels ≤360 µmol/L, before and after the introduction of updated BSR guidelines.**


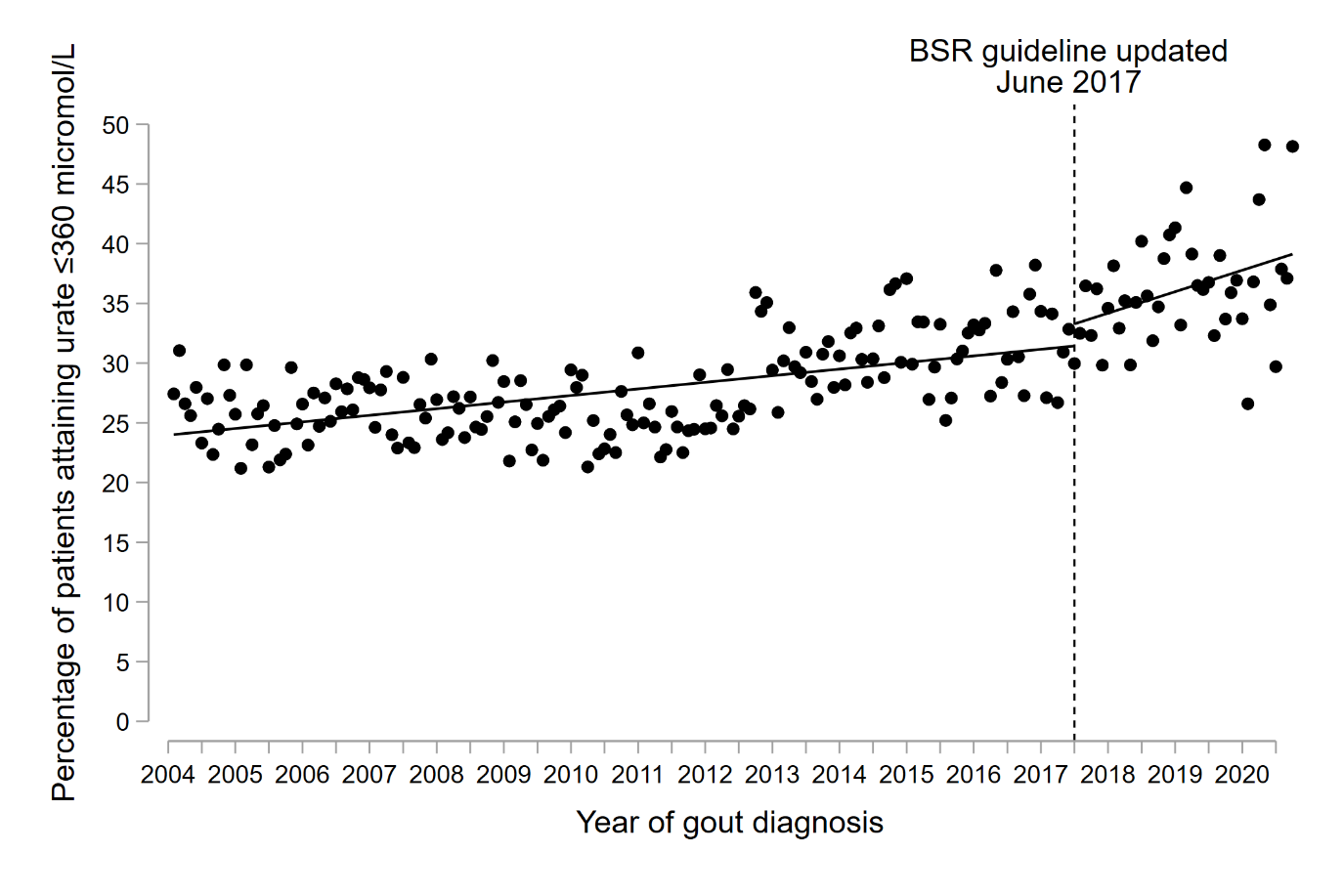


Percentage of patients newly diagnosed with gout who had a serum urate level performed within 12 months of diagnosis (n=65,127) and attained a urate ≤360 µmol/L, comparing trends before and after the introduction of the updated BSR gout management guideline (published in June 2017). Trends were assessed using interrupted time-series analysis, with single time point dots representing monthly average percentages of urate target attainment rates. Rate of improvement post-guideline: 1.80% per year; pre-guideline: 0.55% per year; difference 1.25% per year, 95% CI -0.68 to 3.17, p=0.20.

## **Supplementary Figure S9. Interrupted time-series analysis of attainment of urate levels ≤300 µmol/L, before and after the introduction of updated BSR guidelines.**


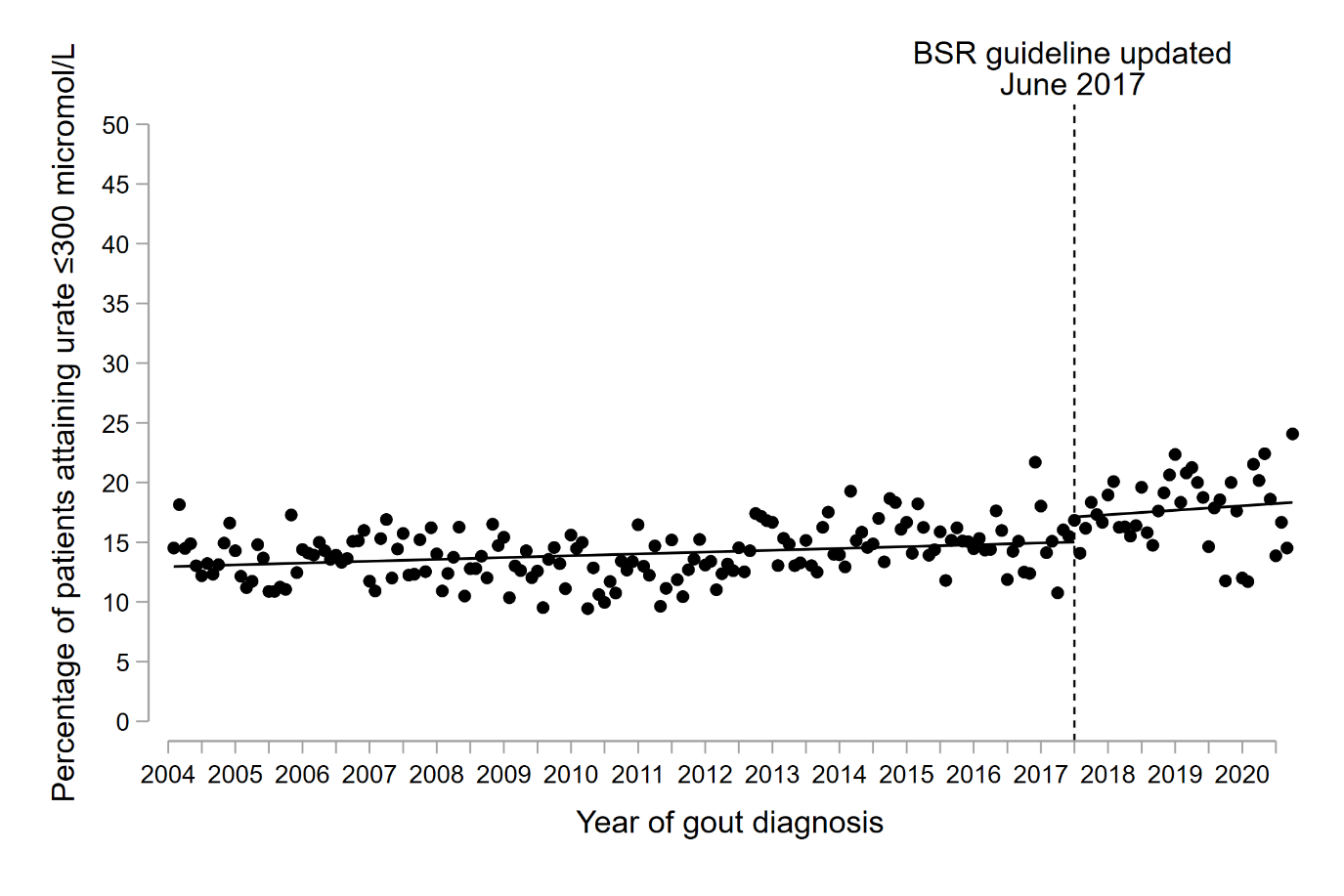


Percentage of patients newly diagnosed with gout who had a serum urate level performed within 12 months of diagnosis (n=65,127) and attained a urate ≤300 µmol/L, comparing trends before and after the introduction of the updated BSR gout management guideline (published in June 2017). Trends were assessed using interrupted time-series analysis, with single time point dots representing monthly average percentages of urate target attainment rates. Rate of improvement post-guideline: 0.37% per year; pre-guideline: 0.15% per year; difference: 0.22% per year, 95% CI -0.99 to 1.43, p=0.72.

## **Supplementary Figure S10. Treat-to-target urate monitoring within 12 months of gout diagnosis, by year of diagnosis**


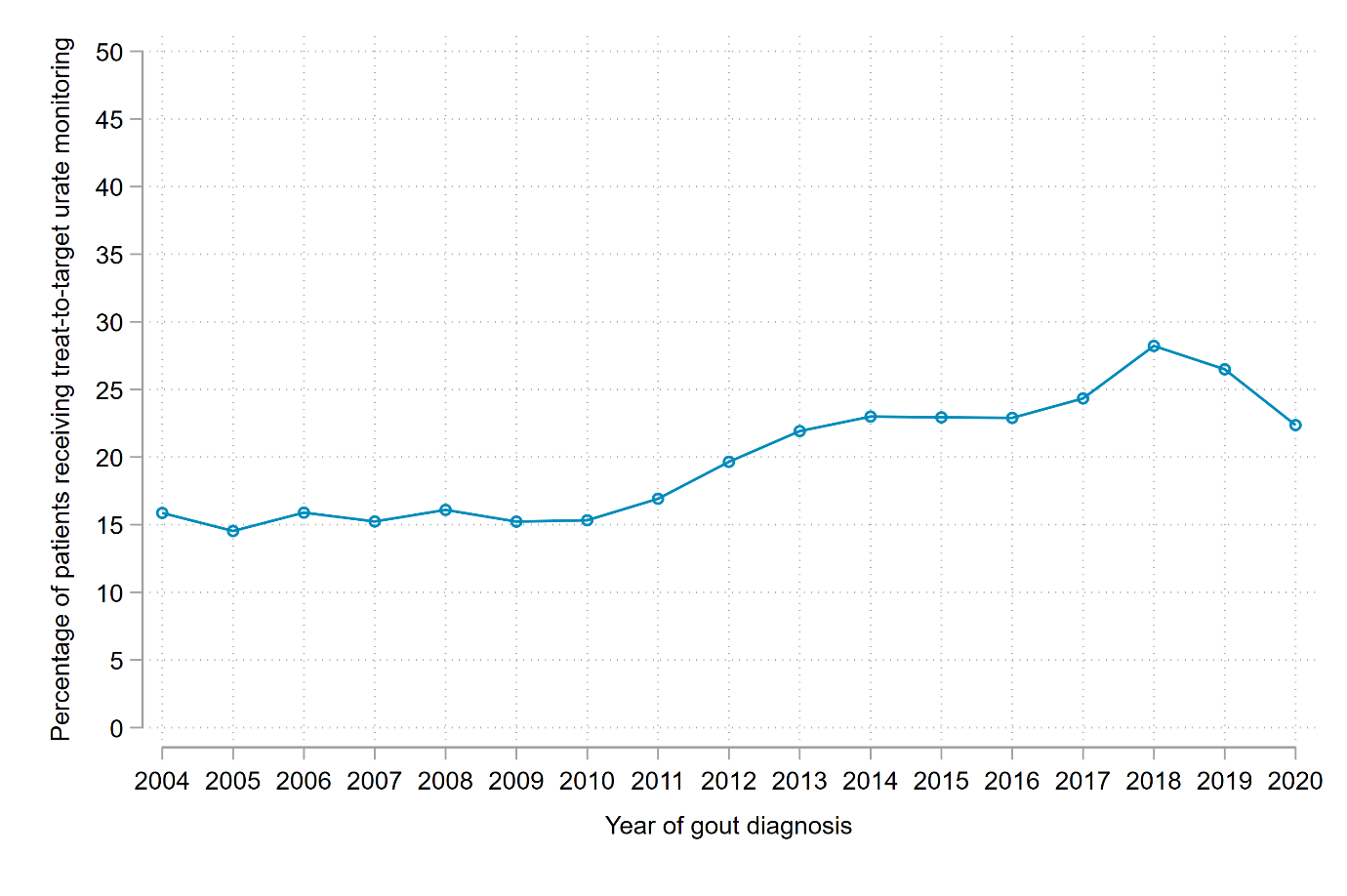


Proportion of newly diagnosed gout patients (n=129,972) who received treat-to-target serum urate monitoring within 12 months of diagnosis, by year of gout diagnosis. Treat-to-target monitoring was defined as two or more urate levels performed within 12 months of diagnosis and/or one or more urate levels ≤300 µmol/L within the same period.

## **Supplementary Figure S11. Initiation of ULT and attainment of urate targets within 24 months of gout diagnosis, separated by year of diagnosis.**


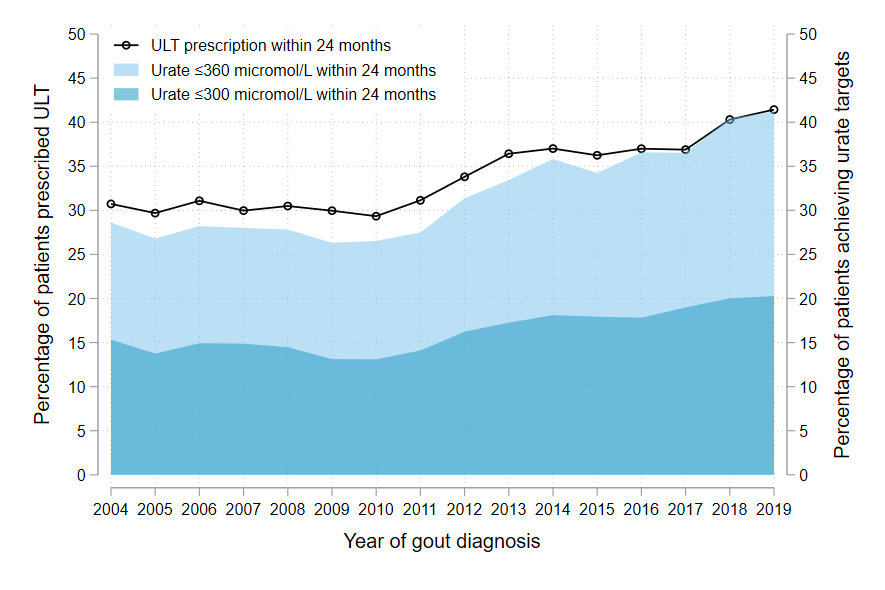


Proportion of patients newly diagnosed with gout, separated by year of diagnosis, who: i) were initiated on urate-lowering therapy (ULT) within 24 months of diagnosis (black line); or ii) had a serum urate performed and attained a level ≤360 µmol/L (light blue) or ≤300 µmol/L (dark blue) within 24 months of diagnosis.

## **Supplementary Figure S12. Treat-to-target urate monitoring within 24 months of gout diagnosis, separated by year of diagnosis**


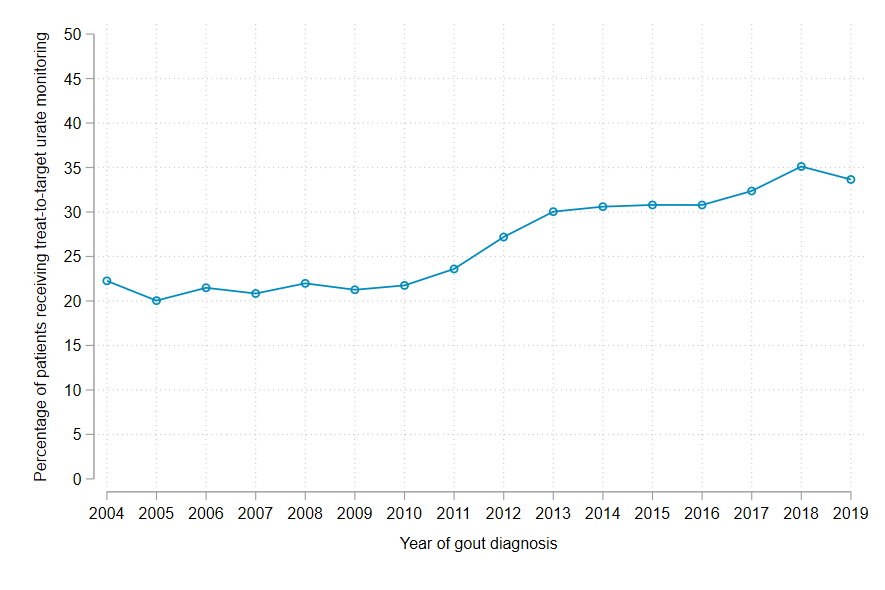


Proportion of newly-diagnosed gout patients who received treat-to-target serum urate monitoring within 24 months of diagnosis, separated by year of gout diagnosis. Treat-to-target monitoring was defined as two or more urate levels performed within 24 months of diagnosis and/or one or more urate levels ≤300 µmol/L within the same period.

## **Supplementary Figure S13. Temporal trends in the initiation of ULT and attainment of urate targets within 12 months of diagnosis for male and female patients with gout.**


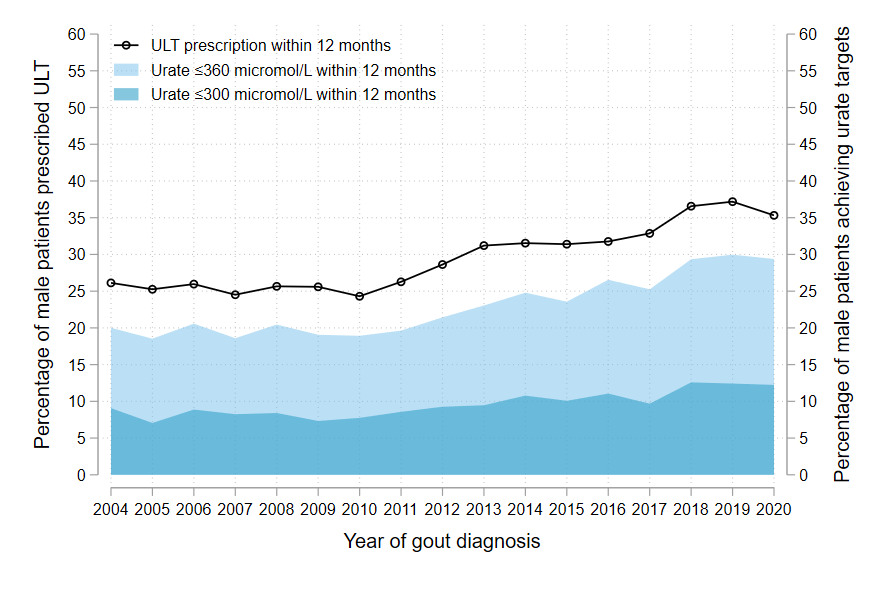


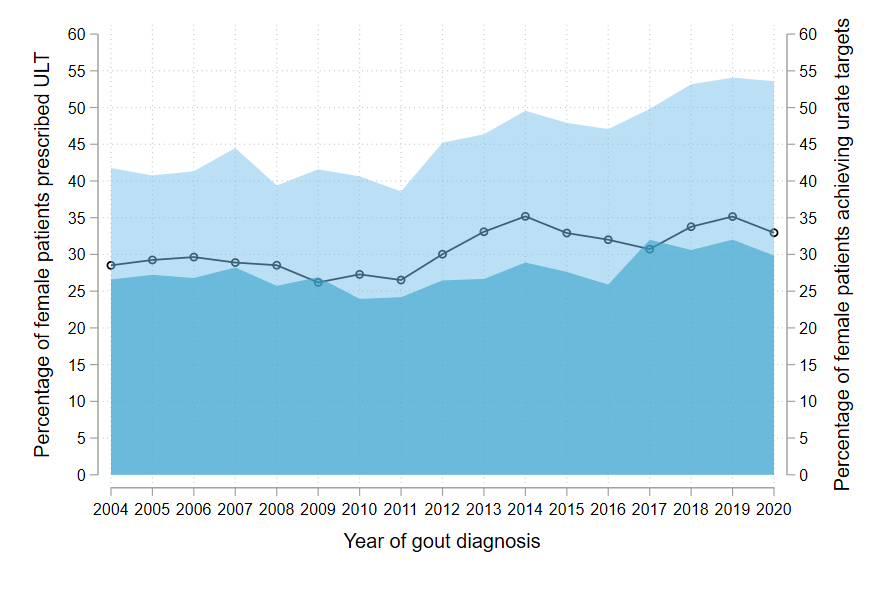


Proportion of male patients (top panel; n=94,610) and female patients (bottom panel; n=35,362) newly diagnosed with gout who: i) were initiated on urate-lowering therapy (ULT) within 12 months of diagnosis (black line); or ii) had a serum urate performed (male: n=47,012; female: n=18,115) and attained a level ≤360 µmol/L (light blue) or ≤300 µmol/L (dark blue) within 12 months of diagnosis, separated by year of diagnosis.

## **Supplementary Figure S14. Impact of multimorbidity on ULT initiation and urate target attainment for male and female patients with gout.**


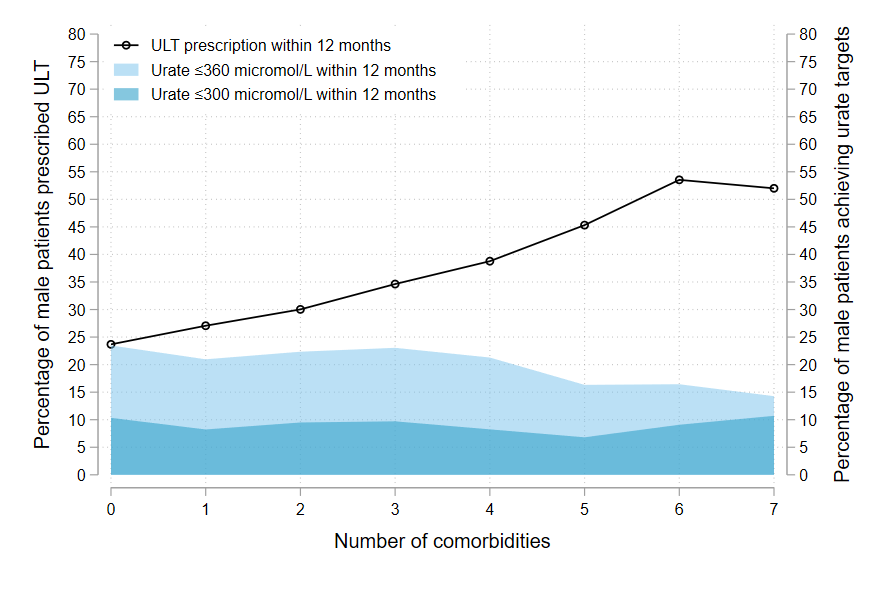


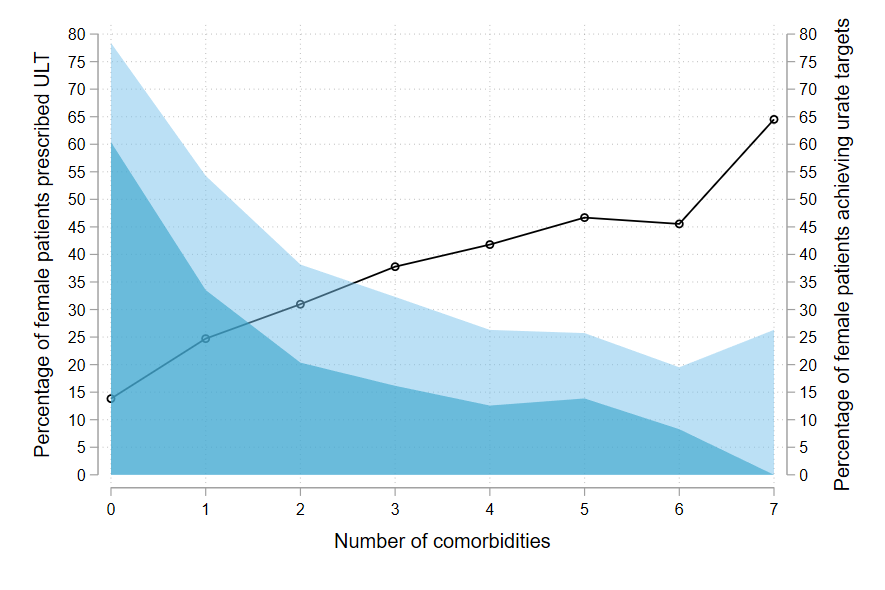


Impact of number of comorbidities at gout diagnosis on the proportion of male patients (top panel; n=94,610) and female patients (bottom panel; n=35,362) who: i) were initiated on urate-lowering therapy (ULT) within 12 months of diagnosis (black line); or ii) had a serum urate performed (male: n=47,012; female: n=18,115) and attained a level ≤360 µmol/L (light blue) or ≤300 µmol/L (dark blue) within 12 months of diagnosis. Comorbidities assessed at baseline were chronic kidney disease stages 3-5, hypertension, diabetes mellitus, ischaemic heart disease, heart failure, previous stroke/transient ischaemic attack and obesity.

## **Supplementary Table S1. Number of patients with newly-diagnosed gout, separated by sex, year of diagnosis and serum urate data availability.**

| **Year of gout**  **diagnosis** | **All Patients** | | | **Patients with post-diagnosis serum urate** | | |
| --- | --- | --- | --- | --- | --- | --- |
|  | **Total** | **Male** | **Female** | **Total** | **Male** | **Female** |
|  | **N=129,972** | **N=94,610** | **N=35,362** | **N=65,127** | **N=47,012** | **N=18,115** |
| 2004 | 7,682 | 5,515 | 2,167 | 3,557 | 2,523 | 1,034 |
| 2005 | 8,889 | 6,540 | 2,349 | 4,081 | 2,972 | 1,109 |
| 2006 | 8,927 | 6,444 | 2,483 | 4,182 | 2,958 | 1,224 |
| 2007 | 8,990 | 6,588 | 2,402 | 4,081 | 2,919 | 1,162 |
| 2008 | 9,517 | 6,906 | 2,611 | 4,465 | 3,206 | 1,259 |
| 2009 | 9,580 | 7,052 | 2,528 | 4,382 | 3,204 | 1,178 |
| 2010 | 9,381 | 6,871 | 2,510 | 4,434 | 3,211 | 1,223 |
| 2011 | 9,716 | 7,155 | 2,561 | 4,730 | 3,473 | 1,257 |
| 2012 | 9,746 | 6,979 | 2,767 | 4,995 | 3,582 | 1,413 |
| 2013 | 9,553 | 6,941 | 2,612 | 5,142 | 3,725 | 1,417 |
| 2014 | 8,376 | 6,037 | 2,339 | 4,541 | 3,254 | 1,287 |
| 2015 | 6,670 | 4,838 | 1,832 | 3,640 | 2,630 | 1,010 |
| 2016 | 5,834 | 4,250 | 1,584 | 3,211 | 2,323 | 888 |
| 2017 | 5,482 | 4,001 | 1,481 | 3,060 | 2,229 | 831 |
| 2018 | 5,043 | 3,678 | 1,365 | 2,986 | 2,153 | 833 |
| 2019 | 4,583 | 3,340 | 1,243 | 2,627 | 1,915 | 712 |
| 2020 | 2,003 | 1,475 | 528 | 1,013 | 735 | 278 |

Number of patients with newly-diagnosed gout in the cohort, separated by year of gout diagnosis and sex. Data are also shown (in the right-hand side of the table) for patients with newly-diagnosed gout who had at least one serum urate level performed within 12 months of diagnosis.

## **Supplementary Table S2. Predictors of attainment of serum urate levels ≤360 µmol/L within 12 months of gout diagnosis.**

| **Variables** | **Odds ratio**  **(univariable)** | **95% CI** | **p-value** | **Odds ratio**  **(multivariable)** | **95% CI** | **p-value** |
| --- | --- | --- | --- | --- | --- | --- |
| Age at diagnosis (per 10-year increase) | 0.97 | (0.96 - 0.98) | <0.001 | 1.11 | (1.10 - 1.13) | <0.001 |
| Female sex | 2.93 | (2.80 - 3.06) | <0.001 | 3.68 | (3.50 - 3.87) | <0.001 |
| Year of gout diagnosis | 1.04 | (1.03 - 1.04) | <0.001 | 1.03 | (1.02 - 1.04) | <0.001 |
| Country: |  |  |  |  |  |  |
| England | Reference |  |  | Reference |  |  |
| Wales | 1.07 | (1.01 - 1.14) | 0.02 | 1.01 | (0.94 - 1.08) | 0.83 |
| Scotland | 1.36 | (1.26 - 1.47) | <0.001 | 1.31 | (1.21 - 1.42) | <0.001 |
| Northern Ireland | 1.48 | (1.30 - 1.68) | <0.001 | 1.48 | (1.30 - 1.69) | <0.001 |
| CKD stages 3-5 | 0.49 | (0.46 - 0.52) | <0.001 | 0.52 | (0.49 - 0.55) | <0.001 |
| Hypertension | 0.70 | (0.67 - 0.73) | <0.001 | 0.92 | (0.88 - 0.97) | <0.001 |
| Diabetes mellitus | 0.98 | (0.92 - 1.04) | 0.48 | 1.23 | (1.16 - 1.31) | <0.001 |
| Ischaemic heart disease | 0.80 | (0.76 - 0.85) | <0.001 | 0.96 | (0.90 - 1.01) | 0.12 |
| Heart failure | 0.60 | (0.56 - 0.65) | <0.001 | 0.82 | (0.75 - 0.89) | <0.001 |
| Previous stroke or TIA | 0.86 | (0.80 - 0.93) | <0.001 | 0.97 | (0.90 - 1.05) | 0.49 |
| Urolithiasis | 1.17 | (1.05 - 1.31) | 0.01 | 1.21 | (1.08 - 1.36) | <0.001 |
| Obesity | 0.73 | (0.70 - 0.76) | <0.001 | 0.75 | (0.73 - 0.78) | <0.001 |
| Current/ex-smoker | 1.10 | (1.05 - 1.14) | <0.001 | 1.13 | (1.09 - 1.18) | <0.001 |
| Alcohol excess | 1.21 | (1.13 - 1.31) | <0.001 | 1.05 | (0.98 - 1.14) | 0.16 |
| Diuretic therapy | 0.51 | (0.49 - 0.54) | <0.001 | 0.63 | (0.60 - 0.67) | <0.001 |

Univariable logistic regression outputs are shown (adjusted for age at diagnosis and sex), in addition to multivariable logistic regression outputs (with adjustment for all predictor variables, including year of diagnosis). Robust standard errors were estimated to account for clustering of patients within practices. CKD: chronic kidney disease; TIA: transient ischaemic attack.

## **Supplementary Table S3. Predictors of time to ULT initiation following new gout diagnoses, using Cox proportional hazards.**

| **Variables** | **Hazard ratio**  **(univariable)** | **95% CI** | **p-value** | **Hazard ratio**  **(multivariable)** | **95% CI** | **p-value** |
| --- | --- | --- | --- | --- | --- | --- |
| Age at diagnosis (per 10-year increase) | 1.02 | (1.01 - 1.03) | <0.001 | 0.91 | (0.91 - 0.92) | <0.001 |
| Female sex | 1.06 | (1.03 - 1.08) | <0.001 | 0.93 | (0.91 - 0.96) | <0.001 |
| Year of gout diagnosis | 1.03 | (1.02 - 1.03) | <0.001 | 1.02 | (1.02 - 1.03) | <0.001 |
| Country: |  |  |  |  |  |  |
| England | Reference |  |  | Reference |  |  |
| Wales | 1.00 | (0.93 - 1.08) | 0.99 | 0.94 | (0.87 – 1.01) | 0.08 |
| Scotland | 1.67 | (1.55 - 1.80) | <0.001 | 1.49 | (1.38 - 1.60) | <0.001 |
| Northern Ireland | 1.65 | (1.46 - 1.87) | <0.001 | 1.49 | (1.33 - 1.68) | <0.001 |
| CKD stages 3-5 | 1.70 | (1.66 - 1.75) | <0.001 | 1.44 | (1.40 - 1.48) | <0.001 |
| Hypertension | 1.29 | (1.26 - 1.32) | <0.001 | 1.05 | (1.03 - 1.08) | <0.001 |
| Diabetes mellitus | 1.24 | (1.21 - 1.28) | <0.001 | 1.02 | (0.99 - 1.05) | 0.22 |
| Ischaemic heart disease | 1.26 | (1.23 - 1.30) | <0.001 | 1.06 | (1.03- 1.09) | <0.001 |
| Heart failure | 1.74 | (1.68 - 1.81) | <0.001 | 1.38 | (1.32 - 1.43) | <0.001 |
| Previous stroke or TIA | 1.11 | (1.07 - 1.16) | <0.001 | 0.98 | (0.95 - 1.02) | 0.43 |
| Urolithiasis | 1.15 | (1.08 - 1.23) | <0.001 | 1.06 | (0.99 - 1.13) | 0.08 |
| Obesity | 1.36 | (1.33 - 1.39) | <0.001 | 1.25 | (1.22 - 1.27) | <0.001 |
| Current or ex-smoker | 1.03 | (1.01 - 1.05) | 0.03 | 0.98 | (0.96 - 1.01) | 0.13 |
| Alcohol excess | 1.22 | (1.16 - 1.27) | <0.001 | 1.08 | (1.03 - 1.13) | <0.001 |
| Diuretic therapy | 1.61 | (1.57 - 1.65) | <0.001 | 1.36 | (1.32 - 1.40) | <0.001 |

Outputs from univariable models (adjusted for age at diagnosis and sex) and multivariable models (adjusting for all predictor variables, including year of diagnosis) are presented. Robust standard errors were estimated to account for clustering of patients within practices. CKD: chronic kidney disease; TIA: transient ischaemic attack.

## **Read codes and definitions**

***Gout***

C34..00 Gout

N023.00 Gouty arthritis

C34z.00 Gout NOS

N023z00 Gouty arthritis NOS

C340.00 Gouty arthropathy

C342.00 Idiopathic gout

C346.00 Acute exacerbation of gout

C345.00 Gout due to impairment of renal function

C344.00 Drug-induced gout

Nyu1700 [X]Other secondary gout

N023700 Gouty arthritis of the ankle and foot

N023300 Gouty arthritis of the forearm

N023600 Gouty arthritis of the lower leg

N023400 Gouty arthritis of the hand

N023x00 Gouty arthritis of multiple sites

N023y00 Gouty arthritis of other specified site

N023100 Gouty arthritis of the shoulder region

N023800 Gouty arthritis of toe

N023200 Gouty arthritis of the upper arm

C343.00 Lead-induced gout

N023000 Gouty arthritis of unspecified site

C34y200 Gouty tophi of other sites

C34y500 Gouty tophi of hand

C34y000 Gouty tophi of ear

C34y100 Gouty tophi of heart

C341000 Gouty nephropathy unspecified

C341z00 Gouty nephropathy NOS

C341.00 Gouty nephropathy

C34y300 Gouty iritis

C34y400 Gouty neuritis

C34yz00 Other specified gouty manifestation NOS

C34y.00 Other specified gouty manifestation

***Chronic kidney disease (CKD)***

Definition - ever or current diagnostic code (see below) for CKD stages 3 to 5, renal failure, dialysis or a renal transplant at the time of index gout diagnosis, and/or two consecutive estimated glomerular filtration rates <60 ml/min/1.73 m^2^ closest to the gout index diagnosis date (assuming they were within 5 years of diagnosis):

1Z12.00 Chronic kidney disease stage 3

1Z13.00 Chronic kidney disease stage 4

1Z15.00 Chronic kidney disease stage 3A

K053.00 Chronic kidney disease stage 3

1Z1E.00 Chronic kidney disease stage 3A without proteinuria

1Z1C.00 Chronic kidney disease stage 3 without proteinuria

1Z14.00 Chronic kidney disease stage 5

1Z16.00 Chronic kidney disease stage 3B

1Z1G.00 Chronic kidney disease stage 3B without proteinuria

1Z1B.00 Chronic kidney disease stage 3 with proteinuria

1Z1D.00 Chronic kidney disease stage 3A with proteinuria

K054.00 Chronic kidney disease stage 4

1Z1F.00 Chronic kidney disease stage 3B with proteinuria

1Z1J.00 Chronic kidney disease stage 4 without proteinuria

1Z1H.00 Chronic kidney disease stage 4 with proteinuria

1Z1T.00 CKD with GFR category G3a & albuminuria category A1

K055.00 Chronic kidney disease stage 5

1Z1X.00 CKD with GFR category G3b & albuminuria category A1

1Z1K.00 Chronic kidney disease stage 5 with proteinuria

1Z1V.00 CKD with GFR category G3a & albuminuria category A2

1Z1Y.00 CKD with GFR category G3b & albuminuria category A2

1Z1C.11 CKD stage 3 without proteinuria

1Z1E.11 CKD stage 3A without proteinuria

1Z1L.00 Chronic kidney disease stage 5 without proteinuria

1Z1H.11 CKD stage 4 with proteinuria

1Z1B.11 CKD stage 3 with proteinuria

1Z1Z.00 CKD with GFR category G3b & albuminuria category A3

1Z1W.00 CKD with GFR category G3a & albuminuria category A3

1Z1J.11 CKD stage 4 without proteinuria

1Z1G.11 CKD stage 3B without proteinuria

1Z1F.11 CKD stage 3B with proteinuria

1Z1D.11 CKD stage 3A with proteinuria

1Z1K.11 CKD stage 5 with proteinuria

1Z1L.11 CKD stage 5 without proteinuria

K050.00 End stage renal failure

7L1A.11 Dialysis for renal failure

K05..00 Chronic renal failure

1Z1..00 Chronic renal impairment

K0D..00 End-stage renal disease

K0E..00 Acute-on-chronic renal failure

K060.11 Impaired renal function

7L1A000 Renal dialysis

7L1A200 Haemodialysis NEC

7L1A100 Peritoneal dialysis

7L1A600 Peritoneal dialysis NEC

7L1A500 Continuous ambulatory peritoneal dialysis

K05..11 Chronic uraemia

7B00.00 Transplantation of kidney

ZV42000 [V]Kidney transplanted

7B00z00 Transplantation of kidney NOS

7B00100 Transplantation of kidney from live donor

8L50.00 Renal transplant planned

7B00200 Transplantation of kidney from cadaver

SP08300 Kidney transplant failure and rejection

TB00111 Renal transplant with complication, without blame

TB00100 Kidney transplant with complication, without blame

SP08H00 Acute rejection of renal transplant

SP08E00 Acute rejection of renal transplant - grade I

7B00400 Allotransplantation kidney from cadaver, heart non-beating

7B00300 Allotransplantation of kidney from cadaver, heart-beating

7B00211 Allotransplantation of kidney from cadaver

7B00111 Allotransplantation of kidney from live donor

SP08F00 Acute rejection of renal transplant - grade II

SP08G00 Acute rejection of renal transplant - grade III

***Hypertension***

Definition - ever or current diagnostic code for hypertension at the time of index gout diagnosis. Absence of the comorbidity was assumed if a diagnostic code was not present:

G2...00 Hypertensive disease

G20..00 Essential hypertension

G2z..00 Hypertensive disease NOS

G201.00 Benign essential hypertension

662O.00 On treatment for hypertension

G20z.11 Hypertension NOS

G672.00 Hypertensive encephalopathy

G202.00 Systolic hypertension

G22..00 Hypertensive renal disease

F421300 Hypertensive retinopathy

G24..00 Secondary hypertension

G2...11 BP - hypertensive disease

G21z011 Cardiomegaly - hypertensive

G20z.00 Essential hypertension NOS

G22z.00 Hypertensive renal disease NOS

G200.00 Malignant essential hypertension

G24z.00 Secondary hypertension NOS

G21zz00 Hypertensive heart disease NOS

G21..00 Hypertensive heart disease

G2y..00 Other specified hypertensive disease

G232.00 Hypertensive heart&renal dis wth (congestive) heart failure

G241000 Secondary benign renovascular hypertension

G233.00 Hypertensive heart and renal disease with renal failure

G22z.11 Renal hypertension

G24z100 Hypertension secondary to drug

G24z000 Secondary renovascular hypertension NOS

G21z.00 Hypertensive heart disease NOS

G240.00 Secondary malignant hypertension

G672.11 Hypertensive crisis

G222.00 Hypertensive renal disease with renal failure

G244.00 Hypertension secondary to endocrine disorders

F404200 Blind hypertensive eye

G220.00 Malignant hypertensive renal disease

G24zz00 Secondary hypertension NOS

G221.00 Benign hypertensive renal disease

G20..11 High blood pressure

14A2.00 H/O: hypertension

G210.00 Malignant hypertensive heart disease

G241z00 Secondary benign hypertension NOS

G211100 Benign hypertensive heart disease with CCF

G211.00 Benign hypertensive heart disease

G241.00 Secondary benign hypertension

G234.00 Hyperten heart&renal dis+both(congestv)heart and renal fail

G240000 Secondary malignant renovascular hypertension

G21z000 Hypertensive heart disease NOS without CCF

G211000 Benign hypertensive heart disease without CCF

G21z100 Hypertensive heart disease NOS with CCF

G231.00 Benign hypertensive heart and renal disease

G23..00 Hypertensive heart and renal disease

G230.00 Malignant hypertensive heart and renal disease

G23z.00 Hypertensive heart and renal disease NOS

Gyu2.00 [X]Hypertensive diseases

G210100 Malignant hypertensive heart disease with CCF

G240z00 Secondary malignant hypertension NOS

G203.00 Diastolic hypertension

L127z00 Pre-eclampsia or eclampsia + pre-existing hypertension NOS

G210000 Malignant hypertensive heart disease without CCF

Gyu2100 [X]Hypertension secondary to other renal disorders

Gyu2000 [X]Other secondary hypertension

G210z00 Malignant hypertensive heart disease NOS

G28..00 Stage 2 hypertension (NICE - Nat Ins for Hth Clin Excl 2011)

G25..11 Stage 1 hypertension

G25..00 Stage 1 hypertension (NICE - Nat Ins for Hth Clin Excl 2011)

G27..00 Hypertension resistant to drug therapy

G26..11 Severe hypertension

G211z00 Benign hypertensive heart disease NOS

G26..00 Severe hypertension (Nat Inst for Health Clinical Ex 2011)

G20..12 Primary hypertension

***Diabetes mellitus***

Definition - ever or current diagnostic code for diabetes mellitus at the time of index gout diagnosis. Absence of the comorbidity was assumed if a diagnostic code was not present:

C10F.00 Type 2 diabetes mellitus

C10..00 Diabetes mellitus

66A4.00 Diabetic on oral treatment

66A3.00 Diabetic on diet only

66AJ.00 Diabetic - poor control

C109.00 Non-insulin dependent diabetes mellitus

C10E.00 Type 1 diabetes mellitus

66A5.00 Diabetic on insulin

F420.00 Diabetic retinopathy

66AI.00 Diabetic - good control

C100112 Non-insulin dependent diabetes mellitus

C109.12 Type 2 diabetes mellitus

C100011 Insulin dependent diabetes mellitus

C108.00 Insulin dependent diabetes mellitus

F420400 Diabetic maculopathy

66AV.00 Diabetic on insulin and oral treatment

C100100 Diabetes mellitus, adult onset, no mention of complication

C100111 Maturity onset diabetes

C101.00 Diabetes mellitus with ketoacidosis

C10FJ00 Insulin treated Type 2 diabetes mellitus

F420600 Non proliferative diabetic retinopathy

C106.00 Diabetes mellitus with neurological manifestation

F420100 Proliferative diabetic retinopathy

C109.11 NIDDM - Non-insulin dependent diabetes mellitus

C108.11 IDDM-Insulin dependent diabetes mellitus

C10FM00 Type 2 diabetes mellitus with persistent microalbuminuria

C106.12 Diabetes mellitus with neuropathy

C10F.11 Type II diabetes mellitus

F372.12 Diabetic neuropathy

1434.00 H/O: diabetes mellitus

C10EM00 Type 1 diabetes mellitus with ketoacidosis

F420200 Preproliferative diabetic retinopathy

C108.12 Type 1 diabetes mellitus

C10FL00 Type 2 diabetes mellitus with persistent proteinuria

C109.13 Type II diabetes mellitus

C109J00 Insulin treated Type 2 diabetes mellitus

C100.00 Diabetes mellitus with no mention of complication

C104.11 Diabetic nephropathy

C107.00 Diabetes mellitus with peripheral circulatory disorder

C100000 Diabetes mellitus, juvenile type, no mention of complication

F420z00 Diabetic retinopathy NOS

C10F600 Type 2 diabetes mellitus with retinopathy

C10FC00 Type 2 diabetes mellitus with nephropathy

66AJz00 Diabetic - poor control NOS

C105.00 Diabetes mellitus with ophthalmic manifestation

C109700 Non-insulin dependent diabetes mellitus - poor control

C10F700 Type 2 diabetes mellitus - poor control

C10E.12 Insulin dependent diabetes mellitus

C100z00 Diabetes mellitus NOS with no mention of complication

C104.00 Diabetes mellitus with renal manifestation

C108900 Insulin dependent diabetes maturity onset

66As.00 Diabetic annual review

66AK.00 Diabetic - cooperative patient

66AJ.11 Unstable diabetes

C10zz00 Diabetes mellitus NOS with unspecified complication

C10FN00 Type 2 diabetes mellitus with ketoacidosis

66AJ100 Brittle diabetes

C10E.11 Type I diabetes mellitus

F420300 Advanced diabetic maculopathy

C10F900 Type 2 diabetes mellitus without complication

C108.13 Type I diabetes mellitus

C10E700 Type 1 diabetes mellitus with retinopathy

F381311 Diabetic amyotrophy

C10G.00 Secondary pancreatic diabetes mellitus

C10EQ00 Type 1 diabetes mellitus with gastroparesis

C10EL00 Type 1 diabetes mellitus with persistent microalbuminuria

C106100 Diabetes mellitus, adult onset, + neurological manifestation

C101z00 Diabetes mellitus NOS with ketoacidosis

C10ED00 Type 1 diabetes mellitus with nephropathy

C10ER00 Latent autoimmune diabetes mellitus in adult

C10E800 Type 1 diabetes mellitus - poor control

F372.11 Diabetic polyneuropathy

C108800 Insulin dependent diabetes mellitus - poor control

C104z00 Diabetes mellitus with nephropathy NOS

C106z00 Diabetes mellitus NOS with neurological manifestation

C10z100 Diabetes mellitus, adult onset, + unspecified complication

66AL.00 Diabetic-uncooperative patient

C103.00 Diabetes mellitus with ketoacidotic coma

F3y0.00 Diabetic mononeuropathy

C10D.00 Diabetes mellitus autosomal dominant type 2

C10C.11 Maturity onset diabetes in youth

13L4.11 Diabetic child

C10FR00 Type 2 diabetes mellitus with gastroparesis

C10EN00 Type 1 diabetes mellitus with ketoacidotic coma

C10z.00 Diabetes mellitus with unspecified complication

C109900 Non-insulin-dependent diabetes mellitus without complication

C10F911 Type II diabetes mellitus without complication

C10FB00 Type 2 diabetes mellitus with polyneuropathy

C10N.00 Secondary diabetes mellitus

L180X00 Pre-existing diabetes mellitus, unspecified

C107.11 Diabetes mellitus with gangrene

C108700 Insulin dependent diabetes mellitus with retinopathy

C10FQ00 Type 2 diabetes mellitus with exudative maculopathy

C10F000 Type 2 diabetes mellitus with renal complications

Cyu2.00 [X]Diabetes mellitus

C10FH00 Type 2 diabetes mellitus with neuropathic arthropathy

C102.00 Diabetes mellitus with hyperosmolar coma

C10FE00 Type 2 diabetes mellitus with diabetic cataract

C109400 Non-insulin dependent diabetes mellitus with ulcer

C108500 Insulin dependent diabetes mellitus with ulcer

L180500 Pre-existing diabetes mellitus, insulin-dependent

C107100 Diabetes mellitus, adult, + peripheral circulatory disorder

C105z00 Diabetes mellitus NOS with ophthalmic manifestation

C106000 Diabetes mellitus, juvenile, + neurological manifestation

C10F200 Type 2 diabetes mellitus with neurological complications

C10FF00 Type 2 diabetes mellitus with peripheral angiopathy

C105100 Diabetes mellitus, adult onset, + ophthalmic manifestation

C10N100 Cystic fibrosis related diabetes mellitus

C10F400 Type 2 diabetes mellitus with ulcer

C101000 Diabetes mellitus, juvenile type, with ketoacidosis

C107.12 Diabetes with gangrene

C10FJ11 Insulin treated Type II diabetes mellitus

C104100 Diabetes mellitus, adult onset, with renal manifestation

C10FD00 Type 2 diabetes mellitus with hypoglycaemic coma

C10A100 Malnutrition-related diabetes mellitus with ketoacidosis

C109600 Non-insulin-dependent diabetes mellitus with retinopathy

C10FE11 Type II diabetes mellitus with diabetic cataract

C10E900 Type 1 diabetes mellitus maturity onset

F420700 High risk proliferative diabetic retinopathy

C10EE00 Type 1 diabetes mellitus with hypoglycaemic coma

C10F100 Type 2 diabetes mellitus with ophthalmic complications

C10C.00 Diabetes mellitus autosomal dominant

C10F711 Type II diabetes mellitus - poor control

C101100 Diabetes mellitus, adult onset, with ketoacidosis

C108400 Unstable insulin dependent diabetes mellitus

C107z00 Diabetes mellitus NOS with peripheral circulatory disorder

C10EP00 Type 1 diabetes mellitus with exudative maculopathy

C10y.00 Diabetes mellitus with other specified manifestation

C109711 Type II diabetes mellitus - poor control

C109712 Type 2 diabetes mellitus - poor control

C109J12 Insulin treated Type II diabetes mellitus

C10z000 Diabetes mellitus, juvenile type, + unspecified complication

C10FG00 Type 2 diabetes mellitus with arthropathy

C10FA00 Type 2 diabetes mellitus with mononeuropathy

C108E00 Insulin dependent diabetes mellitus with hypoglycaemic coma

C10EJ00 Type 1 diabetes mellitus with neuropathic arthropathy

C10F500 Type 2 diabetes mellitus with gangrene

C107400 NIDDM with peripheral circulatory disorder

C10EM11 Type I diabetes mellitus with ketoacidosis

C109C00 Non-insulin dependent diabetes mellitus with nephropathy

C106.13 Diabetes mellitus with polyneuropathy

F420800 High risk non proliferative diabetic retinopathy

C10FC11 Type II diabetes mellitus with nephropathy

C10EA00 Type 1 diabetes mellitus without complication

C107200 Diabetes mellitus, adult with gangrene

C10E400 Unstable type 1 diabetes mellitus

C10F611 Type II diabetes mellitus with retinopathy

C10E500 Type 1 diabetes mellitus with ulcer

L180600 Pre-existing diabetes mellitus, non-insulin-dependent

C10A.00 Malnutrition-related diabetes mellitus

C10E000 Type 1 diabetes mellitus with renal complications

C10FL11 Type II diabetes mellitus with persistent proteinuria

F381300 Myasthenic syndrome due to diabetic amyotrophy

C106y00 Other specified diabetes mellitus with neurological comps

C109312 Type 2 diabetes mellitus with multiple complications

C10y100 Diabetes mellitus, adult, + other specified manifestation

C10F300 Type 2 diabetes mellitus with multiple complications

C10E300 Type 1 diabetes mellitus with multiple complications

C10FD11 Type II diabetes mellitus with hypoglycaemic coma

C10FF11 Type II diabetes mellitus with peripheral angiopathy

C109J11 Insulin treated non-insulin dependent diabetes mellitus

C108000 Insulin-dependent diabetes mellitus with renal complications

C109911 Type II diabetes mellitus without complication

C101y00 Other specified diabetes mellitus with ketoacidosis

C109612 Type 2 diabetes mellitus with retinopathy

C107300 IDDM with peripheral circulatory disorder

C10FB11 Type II diabetes mellitus with polyneuropathy

C106.11 Diabetic amyotrophy

C10D.11 Maturity onset diabetes in youth type 2

C10M.00 Lipoatrophic diabetes mellitus

C102100 Diabetes mellitus, adult onset, with hyperosmolar coma

C10EC00 Type 1 diabetes mellitus with polyneuropathy

C10FP00 Type 2 diabetes mellitus with ketoacidotic coma

C109112 Type 2 diabetes mellitus with ophthalmic complications

C109000 Non-insulin-dependent diabetes mellitus with renal comps

C10FA11 Type II diabetes mellitus with mononeuropathy

C10E100 Type 1 diabetes mellitus with ophthalmic complications

C108A00 Insulin-dependent diabetes without complication

C109611 Type II diabetes mellitus with retinopathy

C10E911 Type I diabetes mellitus maturity onset

C10E200 Type 1 diabetes mellitus with neurological complications

C109E00 Non-insulin depend diabetes mellitus with diabetic cataract

C108100 Insulin-dependent diabetes mellitus with ophthalmic comps

C108D00 Insulin dependent diabetes mellitus with nephropathy

C10E812 Insulin dependent diabetes mellitus - poor control

C10E912 Insulin dependent diabetes maturity onset

C10E711 Type I diabetes mellitus with retinopathy

C109100 Non-insulin-dependent diabetes mellitus with ophthalm comps

C109500 Non-insulin dependent diabetes mellitus with gangrene

C10FG11 Type II diabetes mellitus with arthropathy

C109012 Type 2 diabetes mellitus with renal complications

C10yz00 Diabetes mellitus NOS with other specified manifestation

C10E811 Type I diabetes mellitus - poor control

C10EF00 Type 1 diabetes mellitus with diabetic cataract

C109411 Type II diabetes mellitus with ulcer

C108812 Type 1 diabetes mellitus - poor control

C103z00 Diabetes mellitus NOS with ketoacidotic coma

C10E600 Type 1 diabetes mellitus with gangrene

C108711 Type I diabetes mellitus with retinopathy

C108F00 Insulin dependent diabetes mellitus with diabetic cataract

C108811 Type I diabetes mellitus - poor control

C10E312 Insulin dependent diabetes mellitus with multiple complicat

C108y00 Other specified diabetes mellitus with multiple comps

C109412 Type 2 diabetes mellitus with ulcer

C109212 Type 2 diabetes mellitus with neurological complications

C109200 Non-insulin-dependent diabetes mellitus with neuro comps

C104y00 Other specified diabetes mellitus with renal complications

C10zy00 Other specified diabetes mellitus with unspecified comps

C105000 Diabetes mellitus, juvenile type, + ophthalmic manifestation

C109H00 Non-insulin dependent d m with neuropathic arthropathy

C108712 Type 1 diabetes mellitus with retinopathy

C108C00 Insulin dependent diabetes mellitus with polyneuropathy

C108E11 Type I diabetes mellitus with hypoglycaemic coma

C109B00 Non-insulin dependent diabetes mellitus with polyneuropathy

C109E11 Type II diabetes mellitus with diabetic cataract

C108300 Insulin dependent diabetes mellitus with multiple complicatn

C10EB00 Type 1 diabetes mellitus with mononeuropathy

C108512 Type 1 diabetes mellitus with ulcer

C10E611 Type I diabetes mellitus with gangrene

C109D00 Non-insulin dependent diabetes mellitus with hypoglyca coma

C10E411 Unstable type I diabetes mellitus

C10E712 Insulin dependent diabetes mellitus with retinopathy

C10C.12 Maturity onset diabetes in youth type 1

C10EH00 Type 1 diabetes mellitus with arthropathy

C109C12 Type 2 diabetes mellitus with nephropathy

C10yy00 Other specified diabetes mellitus with other spec comps

C109G00 Non-insulin dependent diabetes mellitus with arthropathy

C109512 Type 2 diabetes mellitus with gangrene

C108511 Type I diabetes mellitus with ulcer

C108600 Insulin dependent diabetes mellitus with gangrene

C10N000 Secondary diabetes mellitus without complication

C102000 Diabetes mellitus, juvenile type, with hyperosmolar coma

C103000 Diabetes mellitus, juvenile type, with ketoacidotic coma

C109E12 Type 2 diabetes mellitus with diabetic cataract

C105y00 Other specified diabetes mellitus with ophthalmic complicatn

C109011 Type II diabetes mellitus with renal complications

C108200 Insulin-dependent diabetes mellitus with neurological comps

C10E112 Insulin-dependent diabetes mellitus with ophthalmic comps

C10E512 Insulin dependent diabetes mellitus with ulcer

C10F311 Type II diabetes mellitus with multiple complications

C10E412 Unstable insulin dependent diabetes mellitus

C10F011 Type II diabetes mellitus with renal complications

C109511 Type II diabetes mellitus with gangrene

C109300 Non-insulin-dependent diabetes mellitus with multiple comps

C103100 Diabetes mellitus, adult onset, with ketoacidotic coma

C102z00 Diabetes mellitus NOS with hyperosmolar coma

C10E511 Type I diabetes mellitus with ulcer

C104000 Diabetes mellitus, juvenile type, with renal manifestation

K01x111 Kimmelstiel - Wilson disease

C109H11 Type II diabetes mellitus with neuropathic arthropathy

C109H12 Type 2 diabetes mellitus with neuropathic arthropathy

C10F111 Type II diabetes mellitus with ophthalmic complications

C109D12 Type 2 diabetes mellitus with hypoglycaemic coma

C108D11 Type I diabetes mellitus with nephropathy

C10FM11 Type II diabetes mellitus with persistent microalbuminuria

C10EG00 Type 1 diabetes mellitus with peripheral angiopathy

C10FN11 Type II diabetes mellitus with ketoacidosis

C108F11 Type I diabetes mellitus with diabetic cataract

C109C11 Type II diabetes mellitus with nephropathy

C108H00 Insulin dependent diabetes mellitus with arthropathy

C10G000 Secondary pancreatic diabetes mellitus without complication

C108412 Unstable type 1 diabetes mellitus

C10E612 Insulin dependent diabetes mellitus with gangrene

Cyu2000 [X]Other specified diabetes mellitus

C109111 Type II diabetes mellitus with ophthalmic complications

C108011 Type I diabetes mellitus with renal complications

C108212 Type 1 diabetes mellitus with neurological complications

C108E12 Type 1 diabetes mellitus with hypoglycaemic coma

C10F411 Type II diabetes mellitus with ulcer

C10E311 Type I diabetes mellitus with multiple complications

C10FH11 Type II diabetes mellitus with neuropathic arthropathy

C108012 Type 1 diabetes mellitus with renal complications

C109B11 Type II diabetes mellitus with polyneuropathy

C103y00 Other specified diabetes mellitus with coma

C109F12 Type 2 diabetes mellitus with peripheral angiopathy

C109211 Type II diabetes mellitus with neurological complications

C10EA12 Insulin-dependent diabetes without complication

C10E012 Insulin-dependent diabetes mellitus with renal complications

C109912 Type 2 diabetes mellitus without complication

C10EQ11 Type I diabetes mellitus with gastroparesis

C109F11 Type II diabetes mellitus with peripheral angiopathy

C108411 Unstable type I diabetes mellitus

C10EA11 Type I diabetes mellitus without complication

C108z00 Unspecified diabetes mellitus with multiple complications

C10A000 Malnutrition-related diabetes mellitus with coma

C107000 Diabetes mellitus, juvenile +peripheral circulatory disorder

C10EE12 Insulin dependent diabetes mellitus with hypoglycaemic coma

C10F511 Type II diabetes mellitus with gangrene

C108J12 Type 1 diabetes mellitus with neuropathic arthropathy

C108B00 Insulin dependent diabetes mellitus with mononeuropathy

C108J11 Type I diabetes mellitus with neuropathic arthropathy

C109A00 Non-insulin dependent diabetes mellitus with mononeuropathy

C10EP11 Type I diabetes mellitus with exudative maculopathy

C10F211 Type II diabetes mellitus with neurological complications

C109D11 Type II diabetes mellitus with hypoglycaemic coma

C108912 Type 1 diabetes mellitus maturity onset

Cyu2300 [X]Unspecified diabetes mellitus with renal complications

C10EC12 Insulin dependent diabetes mellitus with polyneuropathy

L180700 Pre-existing malnutrition-related diabetes mellitus

C109G11 Type II diabetes mellitus with arthropathy

C108211 Type I diabetes mellitus with neurological complications

C109G12 Type 2 diabetes mellitus with arthropathy

C109A11 Type II diabetes mellitus with mononeuropathy

C108H11 Type I diabetes mellitus with arthropathy

C108911 Type I diabetes mellitus maturity onset

C10EN11 Type I diabetes mellitus with ketoacidotic coma

C10EC11 Type I diabetes mellitus with polyneuropathy

C108A11 Type I diabetes mellitus without complication

C108B11 Type I diabetes mellitus with mononeuropathy

C10E111 Type I diabetes mellitus with ophthalmic complications

C10A500 Malnutritn-relat diabetes melitus wth periph circul complctn

C10EF12 Insulin dependent diabetes mellitus with diabetic cataract

C10E212 Insulin-dependent diabetes mellitus with neurological comps

C10ED12 Insulin dependent diabetes mellitus with nephropathy

C10EL11 Type I diabetes mellitus with persistent microalbuminuria

C108112 Type 1 diabetes mellitus with ophthalmic complications

C10FP11 Type II diabetes mellitus with ketoacidotic coma

C108311 Type I diabetes mellitus with multiple complications

***Ischaemic heart disease***

Definition - ever or current diagnostic code for ischaemic heart disease at the time of index gout diagnosis. Absence of the comorbidity was assumed if a diagnostic code was not present:

G3...00 Ischaemic heart disease

G30..00 Acute myocardial infarction

G3...13 IHD - Ischaemic heart disease

G30..15 MI - acute myocardial infarction

G30z.00 Acute myocardial infarction NOS

G3z..00 Ischaemic heart disease NOS

G33..00 Angina pectoris

G307100 Acute non-ST segment elevation myocardial infarction

792..11 Coronary artery bypass graft operations

G340.12 Coronary artery disease

G311500 Acute coronary syndrome

G311.13 Unstable angina

7928.00 Coronary artery bypass graft

G30X000 Acute ST segment elevation myocardial infarction

7929400 Insertion of coronary artery stent

G311100 Unstable angina

14A5.00 H/O: angina pectoris

792..00 Coronary artery operations

G33zz00 Angina pectoris NOS

G308.00 Inferior myocardial infarction NOS

7920y00 Saphenous vein graft replacement of coronary artery OS

793G.00 Perc translumin balloon angioplasty stenting coronary artery

G32..00 Old myocardial infarction

G33z.00 Angina pectoris NOS

G340.11 Triple vessel disease of the heart

G340.00 Coronary atherosclerosis

G33z700 Stable angina

G33z300 Angina on effort

G340000 Single coronary vessel disease

ZV45K11 [V]Presence of coronary artery bypass graft - CABG

G311.00 Preinfarction syndrome

G301z00 Anterior myocardial infarction NOS

7927500 Open angioplasty of coronary artery

G311.11 Crescendo angina

G30..14 Heart attack

G340100 Double coronary vessel disease

7928.11 Percutaneous balloon coronary angioplasty

14A3.00 H/O: myocardial infarct <60

G307.00 Acute subendocardial infarction

7920200 Saphenous vein graft replacement of three coronary arteries

SP07600 Coronary artery bypass graft occlusion

7928z00 Transluminal balloon angioplasty of coronary artery NOS

G311400 Worsening angina

7929500 Insertion of drug-eluting coronary artery stent

G30..12 Coronary thrombosis

ZV45K00 [V]Presence of coronary artery bypass graft

G31y000 Acute coronary insufficiency

G343.00 Ischaemic cardiomyopathy

14A4.00 H/O: myocardial infarct >60

G301.00 Other specified anterior myocardial infarction

G37..00 Cardiac syndrome X

Gyu3.00 [X]Ischaemic heart diseases

G300.00 Acute anterolateral infarction

G302.00 Acute inferolateral infarction

792z.00 Coronary artery operations NOS

G330.00 Angina decubitus

G3...12 Atherosclerotic heart disease

G311200 Angina at rest

G34y100 Chronic myocardial ischaemia

7920300 Saphenous vein graft replacement of four+ coronary arteries

7929300 Rotary blade coronary angioplasty

7920100 Saphenous vein graft replacement of two coronary arteries

G33z400 Ischaemic chest pain

G332.00 Coronary artery spasm

7928000 Percut transluminal balloon angioplasty one coronary artery

7920.11 Saphenous vein graft bypass of coronary artery

G301100 Acute anteroseptal infarction

G30yz00 Other acute myocardial infarction NOS

G32..12 Personal history of myocardial infarction

793G000 Perc translum ball angio insert 1-2 drug elut stents cor art

G341000 Ventricular cardiac aneurysm

G34z000 Asymptomatic coronary heart disease

792Dz00 Other bypass of coronary artery NOS

G304.00 Posterior myocardial infarction NOS

G307000 Acute non-Q wave infarction

7929000 Percutaneous transluminal laser coronary angioplasty

ZV45800 [V]Presence of coronary angioplasty implant and graft

G331.00 Prinzmetal's angina

792D.00 Other bypass of coronary artery

ZV45L00 [V]Status following coronary angioplasty NOS

G305.00 Lateral myocardial infarction NOS

G3y..00 Other specified ischaemic heart disease

G30A.00 Mural thrombosis

G34..00 Other chronic ischaemic heart disease

792B000 Endarterectomy of coronary artery NEC

G311.14 Angina at rest

G303.00 Acute inferoposterior infarction

G33z600 New onset angina

G344.00 Silent myocardial ischaemia

G30..11 Attack - heart

7920000 Saphenous vein graft replacement of one coronary artery

G310.11 Dressler's syndrome

G30..17 Silent myocardial infarction

7A6G100 Peroperative angioplasty

7924.00 Revision of bypass for coronary artery

7921.11 Other autograft bypass of coronary artery

G341.00 Aneurysm of heart

7928100 Percut translum balloon angioplasty mult coronary arteries

G30..16 Thrombosis - coronary

G35..00 Subsequent myocardial infarction

G31y300 Transient myocardial ischaemia

G30y.00 Other acute myocardial infarction

G32..11 Healed myocardial infarction

G34z.00 Other chronic ischaemic heart disease NOS

G342.00 Atherosclerotic cardiovascular disease

7927.00 Open angioplasty of coronary artery

7929.00 Percutaneous transluminal laser coronary angioplasty

G330000 Nocturnal angina

7921.00 Other autograft replacement of coronary artery

7929100 Percut transluminal coronary thrombolysis with streptokinase

7920z00 Saphenous vein graft replacement coronary artery NOS

793G200 Perc translum balloon angioplasty insert 1-2 stents cor art

7928y00 Transluminal balloon angioplasty of coronary artery OS

G33z500 Post infarct angina

792y.00 Other specified operations on coronary artery

793Gz00 Perc translum balloon angioplasty stenting coronary art NOS

G38..00 Postoperative myocardial infarction

14AJ.00 H/O: Angina in last year

G31..00 Other acute and subacute ischaemic heart disease

G30y200 Acute septal infarction

792Dy00 Other specified other bypass of coronary artery

G30..13 Cardiac rupture following myocardial infarction (MI)

G30X.00 Acute transmural myocardial infarction of unspecif site

G34y000 Chronic coronary insufficiency

G341.11 Cardiac aneurysm

7922.11 Allograft bypass of coronary artery

793G100 Perc tran ball angio ins 3 or more drug elut stents cor art

G39..00 Coronary microvascular disease

7921200 Autograft replacement of three coronary arteries NEC

G311300 Refractory angina

7A54500 Rotary blade angioplasty

14AH.00 H/O: Myocardial infarction in last year

7925.00 Connection of mammary artery to coronary artery

G331.11 Variant angina pectoris

G360.00 Haemopericardium/current comp folow acut myocard infarct

G30y000 Acute atrial infarction

G301000 Acute anteroapical infarction

7922.00 Allograft replacement of coronary artery

792A.00 Diagnostic transluminal operations on coronary artery

G309.00 Acute Q-wave infarct

G31y200 Subendocardial ischaemia

7925200 Single anast mammary art to left ant descend coronary art

G311z00 Preinfarction syndrome NOS

G310.00 Postmyocardial infarction syndrome

7923.11 Prosthetic bypass of coronary artery

G31y.00 Other acute and subacute ischaemic heart disease

G312.00 Coronary thrombosis not resulting in myocardial infarction

7927300 Transposition of coronary artery NEC

792C.00 Other replacement of coronary artery

7921100 Autograft replacement of two coronary arteries NEC

G341200 Aneurysm of coronary vessels

7925z00 Connection of mammary artery to coronary artery NOS

7928200 Percut translum balloon angioplasty bypass graft coronary a

7921300 Autograft replacement of four of more coronary arteries NEC

G31yz00 Other acute and subacute ischaemic heart disease NOS

7921000 Autograft replacement of one coronary artery NEC

7922y00 Other specified allograft replacement of coronary artery

G33z200 Syncope anginosa

G30B.00 Acute posterolateral myocardial infarction

G34y.00 Other specified chronic ischaemic heart disease

G501.00 Post infarction pericarditis

G311011 MI - myocardial infarction aborted

G350.00 Subsequent myocardial infarction of anterior wall

G306.00 True posterior myocardial infarction

G351.00 Subsequent myocardial infarction of inferior wall

7926.00 Connection of other thoracic artery to coronary artery

792B.00 Repair of coronary artery NEC

G34yz00 Other specified chronic ischaemic heart disease NOS

G366.00 Thrombosis atrium,auric append&vent/curr comp foll acute MI

ZV45700 [V]Presence of aortocoronary bypass graft

793G300 Percutaneous cor balloon angiop 3 more stents cor art NEC

G330z00 Angina decubitus NOS

G341z00 Aneurysm of heart NOS

G362.00 Ventric septal defect/curr comp fol acut myocardal infarctn

7929z00 Other therapeutic transluminal op on coronary artery NOS

G33z000 Status anginosus

7923.00 Prosthetic replacement of coronary artery

792Bz00 Repair of coronary artery NOS

7925300 Single anastomosis of mammary artery to coronary artery NEC

7925011 LIMA sequential anastomosis

7925311 LIMA single anastomosis

7929y00 Other therapeutic transluminal op on coronary artery OS

7927400 Exploration of coronary artery

G361.00 Atrial septal defect/curr comp folow acut myocardal infarct

792Cz00 Replacement of coronary artery NOS

G381.00 Postoperative transmural myocardial infarction inferior wall

793H000 Percutaneous transluminal balloon dilation cardiac conduit

7922200 Allograft replacement of three coronary arteries

G384.00 Postoperative subendocardial myocardial infarction

7923200 Prosthetic replacement of three coronary arteries

7928300 Percut translum cutting balloon angioplasty coronary artery

Gyu3000 [X]Other forms of angina pectoris

7924200 Revision of bypass for three coronary arteries

G31y100 Microinfarction of heart

7925.11 Creation of bypass from mammary artery to coronary artery

G36..00 Certain current complication follow acute myocardial infarct

7925000 Double anastomosis of mammary arteries to coronary arteries

7929600 Percutaneous transluminal atherectomy of coronary artery

G341100 Other cardiac wall aneurysm

G341300 Acquired atrioventricular fistula of heart

7922300 Allograft replacement of four or more coronary arteries

7A6H400 Percutaneous transluminal angioplasty of vascular graft

7922100 Allograft replacement of two coronary arteries

7925400 Single implantation of mammary artery into coronary artery

7A6H300 Prosthetic graft patch angioplasty

7923300 Prosthetic replacement of four or more coronary arteries

G35X.00 Subsequent myocardial infarction of unspecified site

G38z.00 Postoperative myocardial infarction, unspecified

7924000 Revision of bypass for one coronary artery

7927z00 Other open operation on coronary artery NOS

7921z00 Other autograft replacement of coronary artery NOS

7929111 Percut translum coronary thrombolytic therapy- streptokinase

7924100 Revision of bypass for two coronary arteries

792Ay00 Diagnostic transluminal operation on coronary artery OS

G33z100 Stenocardia

792Az00 Diagnostic transluminal operation on coronary artery NOS

7925y00 Connection of mammary artery to coronary artery OS

792C000 Replacement of coronary arteries using multiple methods

7922z00 Allograft replacement of coronary artery NOS

7923100 Prosthetic replacement of two coronary arteries

792By00 Other specified repair of coronary artery

792Cy00 Other specified replacement of coronary artery

G353.00 Subsequent myocardial infarction of other sites

7925100 Double implant of mammary arteries into coronary arteries

7924z00 Revision of bypass for coronary artery NOS

7921y00 Other autograft replacement of coronary artery OS

7923000 Prosthetic replacement of one coronary artery

7923z00 Prosthetic replacement of coronary artery NOS

7927y00 Other specified other open operation on coronary artery

7929200 Percut translum inject therap subst to coronary artery NEC

G380.00 Postoperative transmural myocardial infarction anterior wall

7924300 Revision of bypass for four or more coronary arteries

G363.00 Ruptur cardiac wall w'out haemopericard/cur comp fol ac MI

7922000 Allograft replacement of one coronary artery

G341111 Mural cardiac aneurysm

G365.00 Rupture papillary muscle/curr comp fol acute myocard infarct

Gyu3600 [X]Subsequent myocardial infarction of unspecified site

G311.12 Impending infarction

G30y100 Acute papillary muscle infarction

7925012 RIMA sequential anastomosis

7926000 Double anastom thoracic arteries to coronary arteries NEC

7926200 Single anastomosis of thoracic artery to coronary artery NEC

7926300 Single implantation thoracic artery into coronary artery NEC

G383.00 Postoperative transmural myocardial infarction unspec site

Gyu3400 [X]Acute transmural myocardial infarction of unspecif site

7924y00 Other specified revision of bypass for coronary artery

7925312 RIMA single anastomosis

7926z00 Connection of other thoracic artery to coronary artery NOS

G364.00 Ruptur chordae tendinae/curr comp fol acute myocard infarct

Gyu3500 [X]Subsequent myocardial infarction of other sites

7924500 Revision of implantation of thoracic artery into heart

***Stroke or transient ischaemic attack***

Definition - ever or current diagnostic code for a stroke or transient ischaemic attack at the time of index gout diagnosis. Absence of the comorbidity was assumed if a diagnostic code was not present:

G66..00 Stroke and cerebrovascular accident unspecified

G65..00 Transient cerebral ischaemia

G65..12 Transient ischaemic attack

G66..11 CVA unspecified

G64z.00 Cerebral infarction NOS

G64..11 CVA - cerebral artery occlusion

G64..00 Cerebral arterial occlusion

G66..13 CVA - Cerebrovascular accident unspecified

G61..00 Intracerebral haemorrhage

G65zz00 Transient cerebral ischaemia NOS

14A7.00 H/O: CVA/stroke

G61..11 CVA - cerebrovascular accid due to intracerebral haemorrhage

14AB.00 H/O: TIA

14A7.12 H/O: stroke

G66..12 Stroke unspecified

G64z.12 Cerebellar infarction

G65..13 Vertebro-basilar insufficiency

G667.00 Left sided CVA

G668.00 Right sided CVA

G64..13 Stroke due to cerebral arterial occlusion

G64..12 Infarction - cerebral

G650.11 Insufficiency - basilar artery

G640.00 Cerebral thrombosis

G65z.00 Transient cerebral ischaemia NOS

G64z200 Left sided cerebral infarction

G6X..00 Cerebrl infarctn due/unspcf occlusn or sten/cerebrl artrs

G64z300 Right sided cerebral infarction

G61..12 Stroke due to intracerebral haemorrhage

G656.00 Vertebrobasilar insufficiency

G61z.00 Intracerebral haemorrhage NOS

G664.00 Cerebellar stroke syndrome

G613.00 Cerebellar haemorrhage

G64z400 Infarction of basal ganglia

G63y000 Cerebral infarct due to thrombosis of precerebral arteries

G663.00 Brain stem stroke syndrome

G651000 Vertebro-basilar artery syndrome

G641.00 Cerebral embolism

G650.00 Basilar artery syndrome

G617.00 Intracerebral haemorrhage, intraventricular

G660.00 Middle cerebral artery syndrome

G640000 Cerebral infarction due to thrombosis of cerebral arteries

G64z000 Brainstem infarction

ZV12512 [V]Personal history of cerebrovascular accident (CVA)

ZV12511 [V]Personal history of stroke

Gyu6400 [X]Other cerebral infarction

G665.00 Pure motor lacunar syndrome

G6W..00 Cereb infarct due unsp occlus/stenos precerebr arteries

G662.00 Posterior cerebral artery syndrome

ZV12D00 [V]Personal history of transient ischaemic attack

G614.00 Pontine haemorrhage

G63y100 Cerebral infarction due to embolism of precerebral arteries

G661.00 Anterior cerebral artery syndrome

G64z.11 Brainstem infarction NOS

G676000 Cereb infarct due cerebral venous thrombosis, nonpyogenic

G65y.00 Other transient cerebral ischaemia

G64z111 Lateral medullary syndrome

G611.00 Internal capsule haemorrhage

G61X000 Left sided intracerebral haemorrhage, unspecified

G61X100 Right sided intracerebral haemorrhage, unspecified

G641000 Cerebral infarction due to embolism of cerebral arteries

G651.00 Vertebral artery syndrome

G666.00 Pure sensory lacunar syndrome

G65z100 Intermittent cerebral ischaemia

G61X.00 Intracerebral haemorrhage in hemisphere, unspecified

G612.00 Basal nucleus haemorrhage

G610.00 Cortical haemorrhage

14AK.00 H/O: Stroke in last year

Gyu6300 [X]Cerebrl infarctn due/unspcf occlusn or sten/cerebrl artrs

G641.11 Cerebral embolus

G653.00 Carotid artery syndrome hemispheric

G64z100 Wallenberg syndrome

Gyu6600 [X]Occlusion and stenosis of other cerebral arteries

G65z000 Impending cerebral ischaemia

G616.00 External capsule haemorrhage

Gyu6G00 [X]Cereb infarct due unsp occlus/stenos precerebr arteries

G618.00 Intracerebral haemorrhage, multiple localized

Gyu6F00 [X]Intracerebral haemorrhage in hemisphere, unspecified

G615.00 Bulbar haemorrhage

Gyu6500 [X]Occlusion and stenosis of other precerebral arteries

G654.00 Multiple and bilateral precerebral artery syndromes

***Heart failure***

Definition - ever or current diagnostic code for heart failure at the time of index gout diagnosis. Absence of the comorbidity was assumed if a diagnostic code was not present:

G580.00 Congestive heart failure

G581.00 Left ventricular failure

G58..00 Heart failure

G580.11 Congestive cardiac failure

G5yy900 Left ventricular systolic dysfunction

G58z.00 Heart failure NOS

G58..11 Cardiac failure

662g.00 New York Heart Association classification - class II

1O1..00 Heart failure confirmed

G581.13 Impaired left ventricular function

585f.00 Echocardiogram shows left ventricular systolic dysfunction

G41z.11 Chronic cor pulmonale

662f.00 New York Heart Association classification - class I

1J60.00 Suspected heart failure

662h.00 New York Heart Association classification - class III

G400.00 Acute cor pulmonale

G5yyA00 Left ventricular diastolic dysfunction

388D.00 New York Heart Assoc classification heart failure symptoms

G581000 Acute left ventricular failure

G580200 Decompensated cardiac failure

14A6.00 H/O: heart failure

G580100 Chronic congestive heart failure

G580.14 Biventricular failure

G580.12 Right heart failure

ZRad.00 New York Heart Assoc classification heart failure symptoms

585g.00 Echocardiogram shows left ventricular diastolic dysfunction

G580000 Acute congestive heart failure

8B29.00 Cardiac failure therapy

G58z.12 Cardiac failure NOS

G554000 Congestive cardiomyopathy

G582.00 Acute heart failure

G580.13 Right ventricular failure

R2y1000 [D]Cardiorespiratory failure

G583.00 Heart failure with normal ejection fraction

662i.00 New York Heart Association classification - class IV

G583.12 Heart failure with preserved ejection fraction

G581.11 Asthma - cardiac

G584.00 Right ventricular failure

G580300 Compensated cardiac failure

Q48y100 Congenital cardiac failure

G1yz100 Rheumatic left ventricular failure

14AM.00 H/O: Heart failure in last year

G580400 Congestive heart failure due to valvular disease

G583.11 HFNEF - heart failure with normal ejection fraction

G21z100 Hypertensive heart disease NOS with CCF

G211100 Benign hypertensive heart disease with CCF

G5yyB00 Right ventricular diastolic dysfunction

G210.00 Malignant hypertensive heart disease

G232.00 Hypertensive heart&renal dis wth (congestive) heart failure

G230.00 Malignant hypertensive heart and renal disease

G554011 Congestive obstructive cardiomyopathy

G234.00 Hyperten heart&renal dis+both(congestv)heart and renal fail

G557100 Beriberi heart disease

G210100 Malignant hypertensive heart disease with CCF

SP11111 Heart failure as a complication of care

G5y4z00 Post cardiac operation heart failure NOS

***Obesity***

Definition – recorded body mass index ≥30 kg/m^2^ on the reading closest to the index gout diagnosis date (assuming this reading was within 5 years before or after the diagnosis date).

***Urolithiasis***

Definition - ever or current diagnostic code for urolithiasis at the time of index gout diagnosis. Urolithiasis was assumed not present in the absence of a diagnostic code:

K120.12 Renal calculus

K120.13 Renal stone

K120.00 Calculus of kidney

7B0B.00 Extracorporeal shockwave lithotripsy for renal calculus

K121.12 Ureteric stone

K121.11 Ureteric calculus

K12..00 Calculus of kidney and ureter

C341111 Renal stone - uric acid

K121.00 Calculus of ureter

7B07.12 Percutaneous lithotripsy of renal calculus

K12z.00 Urinary calculus NOS

K120z00 Renal calculus NOS

K140.11 Bladder stone

7B18.00 Ureteroscopic operations for ureteric calculus

4G4..11 O/E: kidney stone

K140.00 Bladder calculus

7B1C.00 Extracorporeal shockwave lithotripsy of ureteric calculus

7B07.00 Percutaneous renal stone surgery

K120000 Staghorn calculus

K120.11 Nephrolithiasis NOS

4G4..00 O/E: renal calculus

7B18000 Ureteroscopic laser lithotripsy of ureteric calculus

K141.00 Calculus in urethra

K12..12 Urinary calculus

7B18200 Ureteroscopic extraction of ureteric calculus

7B18100 Other ureteroscopic fragmentation of ureteric calculus

4G6..00 O/E - ureteric calculus

7B05000 Unspecified open removal of calculus from kidney

7B19000 Cystoscopic laser lithotripsy of ureteric calculus

K12..11 Kidney calculus

7B19.00 Cystoscopic removal of ureteric calculus

7B17111 Other nephroscopic lithotripsy of ureteric calculus

C341100 Uric acid nephrolithiasis

7B0B000 ESWL for renal calculus of unspecified size

7B17000 Nephroscopic laser lithotripsy of ureteric calculus

7B18011 Ureteroscopic laser fragmentation of ureteric calculus

14D3.00 H/O: urinary stone

7B0B.11 Extracorporeal fragmentation of renal calculus

7B29400 Electrokinetic lithotripsy of bladder calculus

7B07211 Endoscopic laser fragmentation of renal calculus

K140z00 Bladder calculus NOS

7B0Bz00 Extracorporeal shockwave lithotripsy for renal calculus NOS

7B29100 Other endoscopic extraction of bladder calculus

7B25000 Open removal of bladder calculus

7B19200 Cystoscopic extraction of ureteric calculus

7B07200 Nephroscopy and laser lithotripsy of renal calculus

7B17100 Other nephroscopic fragmentation of ureteric calculus

7B19212 Dormia basket extraction of ureteric calculus

4G8..00 O/E - bladder calculus

7B07400 Endoscopic extraction of calculus of kidney nec

7B19211 Basket extraction of ureteric calculus

7B07z00 Percutaneous renal stone surgery NOS

7B42300 Open urethrotomy and removal of calculus

K122.00 Calculus of kidney with calculus of ureter

7B43900 Endoscopic removal of urethral calculus

K14..00 Lower urinary tract calculus

7B07.11 Nephroscopic percutaneous lithotripsy of renal calculus

7B07011 Endoscopic ultrasound fragmentation of renal calculus

7B0By00 Extracorporeal shockwave lithotripsy for renal calculus OS

7B1Cz00 Extracorporeal shockwave lithotripsy ureteric calculus NOS

7B19400 Cystoscopic dilation of ureter for drainage of calculus

K14z.00 Lower urinary tract calculus NOS

4G4Z.00 O/E: renal stone NOS

7B19z00 Cystoscopic removal of ureteric calculus NOS

7B0B100 ESWL for renal calculus less than 2 cm in diameter

7B19100 Other cystoscopic fragmentation of ureteric calculus

7B07000 Nephroscopy and ultrasound lithotripsy of renal calculus

7B17200 Nephroscopic extraction of ureteric calculus

7B17011 Nephroscopic laser fragmentation of ureteric calculus

7B1C000 Extracorp shockwave lithotripsy of unspec ureteric calculus

7B2B400 Removal of bladder calculus by urethral catheter suction

7B1Cy00 Extracorporeal shockwave lithotripsy of ureteric calculus OS

7B07y00 Other specified percutaneous renal stone surgery

7B1C100 Extracorporeal shockwave therapy for stone in upper ureter

K14y.00 Other lower urinary tract calculus

4G82.11 Uric acid bladder stone

7B1C300 Extracorporeal shockwave lithotripsy stone in lower ureter

7B07100 Nephroscopy & electrohydraulic lithotripsy of renal calculus

4G43.00 O/E: uric acid renal calculus

7B0B200 ESWL for renal calculus 2 cm or more in diameter

7B19300 Cystoscopic catheter drainage for ureteric calculus

K140100 Other calculus in bladder

4G81.11 Oxalate bladder stone

7B19y00 Other specified cystoscopic removal of ureteric calculus

7B1C200 Extracorporeal shockwave lithotripsy for stone in mid-ureter

4G42.11 Phosphate kidney stone

Kyu3100 [X]Calculus of urinary tract in other diseases CE

4GA5.00 Calculus = uric acid

Kyu3000 [X]Other lower urinary tract calculus

***Alcohol excess***

Definition - ever or current diagnostic code for alcohol excess or an alcohol-related problem at the time of index gout diagnosis. Absence of the comorbidity was assumed if a diagnostic code was not present:

E23..00 Alcohol dependence syndrome

136K.00 Alcohol intake above recommended sensible limits

E23..12 Alcohol problem drinking

8BA8.00 Alcohol detoxification

E250.00 Nondependent alcohol abuse

E23..11 Alcoholism

E250000 Nondependent alcohol abuse, unspecified

E01y000 Alcohol withdrawal syndrome

136T.00 Harmful alcohol use

136S.00 Hazardous alcohol use

136P.00 Heavy drinker

66e..00 Alcohol disorder monitoring

J612.00 Alcoholic cirrhosis of liver

J613.00 Alcoholic liver damage unspecified

E23z.00 Alcohol dependence syndrome NOS

66e0.00 Alcohol abuse monitoring

Eu10800 [X]Alcohol withdrawal-induced seizure

E250z00 Nondependent alcohol abuse NOS

J610.00 Alcoholic fatty liver

E230.00 Acute alcoholic intoxication in alcoholism

9k12.00 Alcohol misuse - enhanced service completed

136W.00 Alcohol misuse

J611.00 Acute alcoholic hepatitis

E231.00 Chronic alcoholism

J617.00 Alcoholic hepatitis

136Q.00 Very heavy drinker

Eu10211 [X]Alcohol addiction

E250200 Nondependent alcohol abuse, episodic

E231z00 Chronic alcoholism NOS

8H35.00 Admitted to alcohol detoxification centre

R103.00 [D]Alcohol blood level excessive

Eu10212 [X]Chronic alcoholism

E011000 Korsakov's alcoholic psychosis

E010.12 Delirium tremens

Eu10.00 [X]Mental and behavioural disorders due to use of alcohol

E010.00 Alcohol withdrawal delirium

Eu10411 [X]Delirium tremens, alcohol induced

Eu10200 [X]Mental and behav dis due to use alcohol: dependence syndr

ZV11300 [V]Personal history of alcoholism

F375.00 Alcoholic polyneuropathy

G555.00 Alcoholic cardiomyopathy

E010.11 DTs - delirium tremens

E250100 Nondependent alcohol abuse, continuous

E012.11 Alcoholic dementia NOS

J671000 Alcohol-induced chronic pancreatitis

Eu10000 [X]Mental & behav dis due to use alcohol: acute intoxication

Eu10711 [X]Alcoholic dementia NOS

E01..00 Alcoholic psychoses

Eu10514 [X]Alcoholic psychosis NOS

F11x011 Alcoholic encephalopathy

E231200 Episodic chronic alcoholism

E011200 Wernicke-Korsakov syndrome

E231100 Continuous chronic alcoholism

Eu10511 [X]Alcoholic hallucinosis

G852300 Oesophageal varices in alcoholic cirrhosis of the liver

J670800 Alcohol-induced acute pancreatitis

J613000 Alcoholic hepatic failure

Eu10100 [X]Mental and behav dis due to use of alcohol: harmful use

E231300 Chronic alcoholism in remission

63C7.00 Maternal alcohol abuse

E230z00 Acute alcoholic intoxication in alcoholism NOS

E013.00 Alcohol withdrawal hallucinosis

Eu10300 [X]Mental and behav dis due to use alcohol: withdrawal state

E231000 Unspecified chronic alcoholism

Eu10712 [X]Chronic alcoholic brain syndrome

Z191.00 Alcohol detoxification

E01y.00 Other alcoholic psychosis

Eu10500 [X]Mental & behav dis due to use alcohol: psychotic disorder

J612000 Alcoholic fibrosis and sclerosis of liver

E230000 Acute alcoholic intoxication, unspecified, in alcoholism

E012.00 Other alcoholic dementia

E015.00 Alcoholic paranoia

E250300 Nondependent alcohol abuse in remission

J617000 Chronic alcoholic hepatitis

E230300 Acute alcoholic intoxication in remission, in alcoholism

Eu10600 [X]Mental and behav dis due to use alcohol: amnesic syndrome

E01z.00 Alcoholic psychosis NOS

E230200 Episodic acute alcoholic intoxication in alcoholism

Eu10400 [X]Men & behav dis due alcohl: withdrawl state with delirium

E011100 Korsakov's alcoholic psychosis with peripheral neuritis

E011.00 Alcohol amnestic syndrome

E014.00 Pathological alcohol intoxication

E012000 Chronic alcoholic brain syndrome

E230100 Continuous acute alcoholic intoxication in alcoholism

Eu10700 [X]Men & behav dis due alcoh: resid & late-onset psychot dis

Eu10z00 [X]Ment & behav dis due use alcohol: unsp ment & behav dis

Eu10y00 [X]Men & behav dis due to use alcohol: oth men & behav dis

E01yz00 Other alcoholic psychosis NOS

E011z00 Alcohol amnestic syndrome NOS

***Smoking status***

Definition - ever or current diagnostic code for being an ex-smoker or current smoker at the time of index gout diagnosis:

137S.00 Ex smoker

8CAL.00 Smoking cessation advice

137P.00 Cigarette smoker

137..00 Tobacco consumption

137R.00 Current smoker

137K.00 Stopped smoking

137P.11 Smoker

137F.00 Ex-smoker - amount unknown

137G.00 Trying to give up smoking

137A.00 Ex-heavy smoker (20-39/day)

1372.11 Occasional smoker

137M.00 Rolls own cigarettes

745H.00 Smoking cessation therapy

8HTK.00 Referral to stop-smoking clinic

13p0.00 Negotiated date for cessation of smoking

137J.00 Cigar smoker

8IEM.00 Smoking cessation drug therapy declined

137H.00 Pipe smoker

137..11 Smoker - amount smoked

137B.00 Ex-very heavy smoker (40+/day)

8IEK.00 Smoking cessation programme declined

137d.00 Not interested in stopping smoking

13p5.00 Smoking cessation programme start date

137Z.00 Tobacco consumption NOS

137X.00 Cigarette consumption

67H6.00 Brief intervention for smoking cessation

137c.00 Thinking about stopping smoking

8IEo.00 Referral to smoking cessation service declined

137b.00 Ready to stop smoking

137C.00 Keeps trying to stop smoking

9NS0200 Referral for smoking cessation service offered

8T08.00 Referral to smoking cessation service

745H400 Smoking cessation drug therapy

137N.00 Ex pipe smoker

137Q.00 Smoking started

137a.00 Pipe tobacco consumption

ZG23300 Advice on smoking

137Q.11 Smoking restarted

137O.00 Ex cigar smoker

E251.00 Tobacco dependence

8CdB.00 Stop smoking service opportunity signposted

137Y.00 Cigar consumption

137V.00 Smoking reduced

13p5000 Practice based smoking cessation programme start date

9ko..11 Current smoker annual review

137m.00 Rolls own cigarettes

137K000 Recently stopped smoking

745Hy00 Other specified smoking cessation therapy

8HBM.00 Stop smoking face to face follow-up

137e.00 Smoking restarted

E251z00 Tobacco dependence NOS

9ko..00 Current smoker annual review - enhanced services admin

ZV4K000 [V]Tobacco use

E251100 Tobacco dependence, continuous

E251000 Tobacco dependence, unspecified

1V08.00 Smokes drugs in cigarette form

8B2B.00 Nicotine replacement therapy

745H000 Nicotine replacement therapy using nicotine patches

8B3Y.00 Over the counter nicotine replacement therapy

745H200 Nicotine replacement therapy using nicotine inhalator

E023.00 Nicotine withdrawal

745H100 Nicotine replacement therapy using nicotine gum

8I39.00 Nicotine replacement therapy refused

745H300 Nicotine replacement therapy using nicotine lozenges

8BP3.00 Nicotine replacement therapy provided by community pharmacis

SMC..00 Toxic effect of tobacco and nicotine

***Diuretic therapy at baseline***

Definition - prescription issued for a diuretic medication (furosemide, bendroflumethiazide, spironolactone, bumetanide, indapamide, hydrochlorothiazide, eplerenone, metolazone, amiloride, torasemide, chlortalidone, benzthiazide or xipamide) within 4 months of the index gout diagnosis date.
